# Neuroinflammation in Alzheimer’s disease: insights from peripheral immune cells

**DOI:** 10.1186/s12979-024-00445-0

**Published:** 2024-06-14

**Authors:** Qiang Zhang, Guanhu Yang, Yuan Luo, Lai Jiang, Hao Chi, Gang Tian

**Affiliations:** 1 Department of Laboratory Medicine, Southwest Medical University, Luzhou, China; 2 Department of Specialty Medicine, Ohio University, Athens, OH USA; 3 Clinical Medical College, Southwest Medical University, Luzhou, China; 4 Department of Laboratory Medicine, Engineering Technology Research Center of Molecular Diagnosis of Clinical Diseases, Molecular Diagnosis of Clinical Diseases Key Laboratory of Luzhou, The Affiliated Hospital of Southwest Medical University, Sichuan, 646000 China

**Keywords:** Alzheimer’s disease, Neuroinflammation, Neutrophils, T lymphocytes, B lymphocytes, NK cells

## Abstract

Alzheimer’s disease (AD) is a serious brain disorder characterized by the presence of beta-amyloid plaques, tau pathology, inflammation, neurodegeneration, and cerebrovascular dysfunction. The presence of chronic neuroinflammation, breaches in the blood-brain barrier (BBB), and increased levels of inflammatory mediators are central to the pathogenesis of AD. These factors promote the penetration of immune cells into the brain, potentially exacerbating clinical symptoms and neuronal death in AD patients. While microglia, the resident immune cells of the central nervous system (CNS), play a crucial role in AD, recent evidence suggests the infiltration of cerebral vessels and parenchyma by peripheral immune cells, including neutrophils, T lymphocytes, B lymphocytes, NK cells, and monocytes in AD. These cells participate in the regulation of immunity and inflammation, which is expected to play a huge role in future immunotherapy. Given the crucial role of peripheral immune cells in AD, this article seeks to offer a comprehensive overview of their contributions to neuroinflammation in the disease. Understanding the role of these cells in the neuroinflammatory response is vital for developing new diagnostic markers and therapeutic targets to enhance the diagnosis and treatment of AD patients.

## Introduction

According to the most recent report from Alzheimer’s Disease (AD) International, the number of individuals with dementia worldwide is projected to increase from 50 million in 2019 to 152 million by 2050. Furthermore, the annual cost of dementia is estimated to increase from $1 trillion in 2019 to $2 trillion in 2030, and it will further increase to $9.12 trillion by 2050 [1]. According to the report “Global status report on the public health response to dementia” from WHO in 2021, AD is the most common form of dementia, accounting for approximately 60–70% of all cases. This neurodegenerative disorder progresses over time, resulting in memory and cognitive issues [2]. AD is strongly associated with aging, with 10% of people aged 65 and over and 32% of those aged 85 and over having been diagnosed with the condition [3]. Apart from age, genetic factors are major risk factors for AD [4], particularly the APOEε4 gene, which contributes to the development and progression of AD by influencing lipid metabolism [5], reducing amyloid-beta (Aβ) clearance [6], exacerbating neuroinflammation [7], and affecting synaptic function and neuroplasticity [8]. In addition, traumatic brain injury [9], stroke [10], various tumors [11], viral infections [12], diabetes [13], hypertension [14], cardiovascular disease [13], obstructive sleep apnea [15], and obesity can also contribute to the onset of AD.

**Fig. 1:**
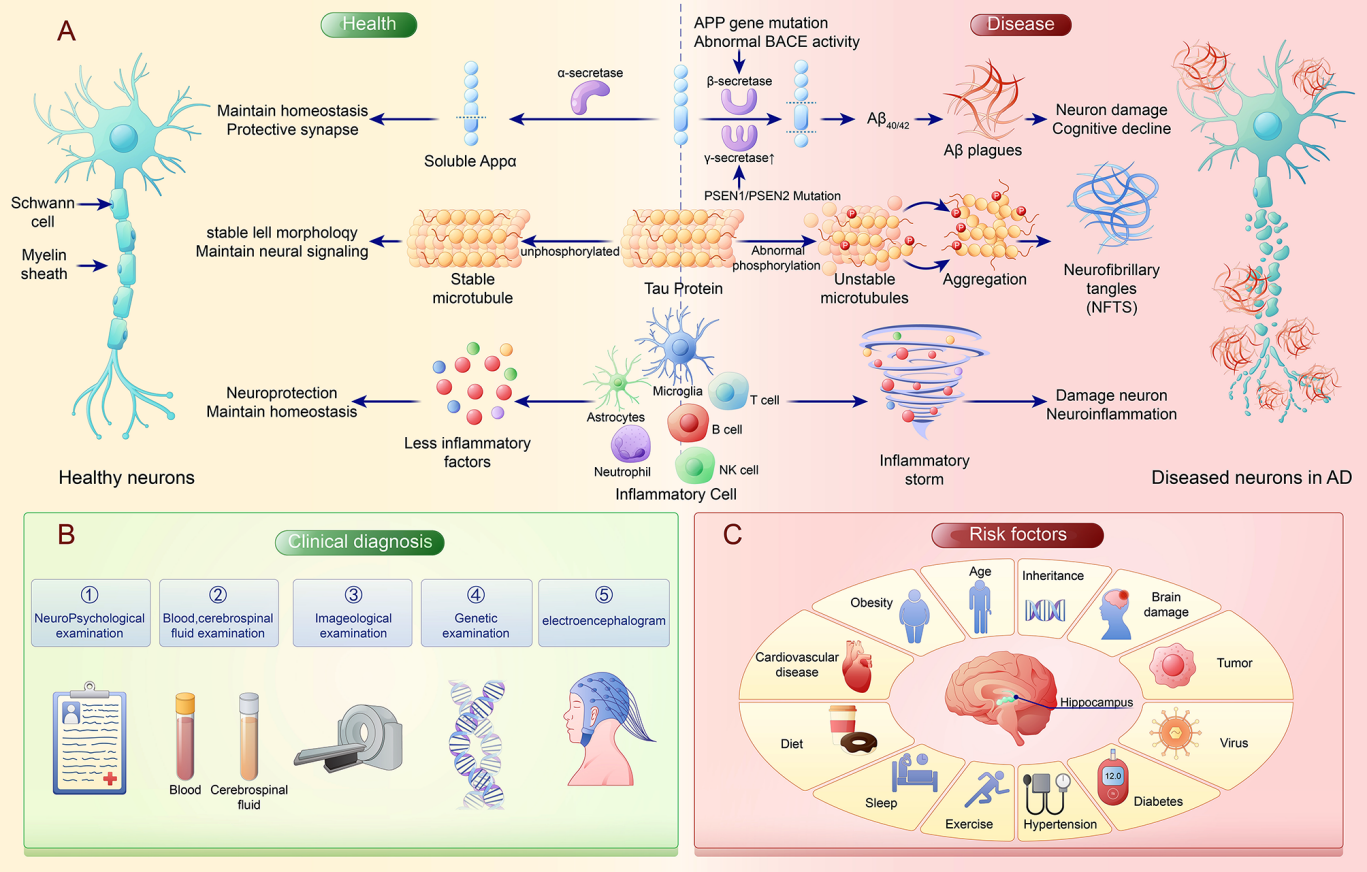
The pathological mechanism, clinical diagnosis, and potential risk factors of Alzheimer’s disease. (**A**) Alzheimer’s Disease (AD) is characterized by the accumulation of Aβ plaques and hyperphosphorylated tau protein in the brain, leading to neuronal damage and the formation of neurofibrillary tangles. In addition, neuroinflammation caused by cytokine storm triggered by the joint action of microglia, astrocytes, and peripheral immune cells such as neutrophils, lymphocytes, and NK cells plays an important role in the disease progression of AD. (**B**) The diagnosis of AD typically involves a combination of neuropsychological assessments, biomarker analysis, neuroimaging studies, and electroencephalography. (**C**) The risk of developing AD is influenced by various factors, including age, genetic predisposition, weight management, sleep quality, mental health, and physical activity. The interaction of these factors determines an individual’s likelihood of developing AD

AD is characterized primarily by the formation of Aβ plaques and the tangling of neurofibrils. Enzymatic digestion of the amyloid precursor protein (APP) produces various lengths of Aβ, with Aβ_1–42_ being more prone to aggregation compared to the more soluble Aβ_1–40_, resulting in cytotoxic effects [16–18]. The Arctic mutation (E693G) and mutations in genes coding for PS (Presenilin) 1 and PS2 proteins (PSEN1 and PSEN2) are linked to abnormal APP metabolism and early-onset familial AD [19–21], while the APOE ε4 allele is identified as a risk factor associated with increased Aβ accumulation [22, 23]. When clearance mechanisms fail or APP metabolism is disrupted, higher Aβ production results in plaque formation, impeding neuronal communication and causing functional decline and cell death [24]. Additionally, in healthy conditions, the tau protein maintains neuronal structure and function [25]. However, excessive phosphorylation of tau leads to the formation of neurofibrillary tangles, which disrupt neuronal metabolism and signal transmission, ultimately causing neuronal death and cognitive impairment [26]. Subsequent pathological changes include cerebral amyloid angiopathy, neuronal loss, and synaptic dysfunction [27–29]. Research has indicated that neuroinflammatory reactions are a key factor in the progression of AD [30]. It is widely recognized that in specific hippocampal regions of the brain, microglia and astrocytes are the primary cells involved in the neuroinflammatory response [31]. Furthermore, the interaction between complement and these two types of cells [32], as well as the formation of inflammasomes, particularly the NLRP3 inflammasome [33], contributes to exacerbating neuroinflammation in the brain by promoting Aβ and tau pathology [34], and inducing the release of IL (Interleukin) -1β and IL-18 [35]. This ultimately leads to the disruption of the BBB, a key pathological feature of AD [36]. Besides, a previous study has shown that elevated levels of pro-inflammatory cytokines and chemokines in the peripheral system contribute to of the advancement of AD [37]. The pro-inflammatory environment can trigger the innate and adaptive immune systems, resulting in the recruitment of peripheral immune cells into brain tissue through the weakened BBB. This may be involved in the pathological progression of AD. AD has the potential to induce a systemic inflammatory reaction, leading to the activation of peripheral immune cells such as neutrophils, T lymphocytes, B lymphocytes, and NK cells [38–42]. This can result in the formation of a cytokine storm, damaging neurons. Figure 1 summarizes the pathological features and mechanisms of AD, methods for clinical diagnosis, and risk factors for the development of AD. In this review, we aim to offer a comprehensive overview of the contributions of neutrophils, T lymphocytes, B lymphocytes, NK cells, and monocytes to neuroinflammation in AD. Furthermore, it delves into the pathogenesis of AD to enhance the diagnosis and management of the disease in the future.

## Peripheral immune cells and neuroinflammation in AD

**Fig. 2:**
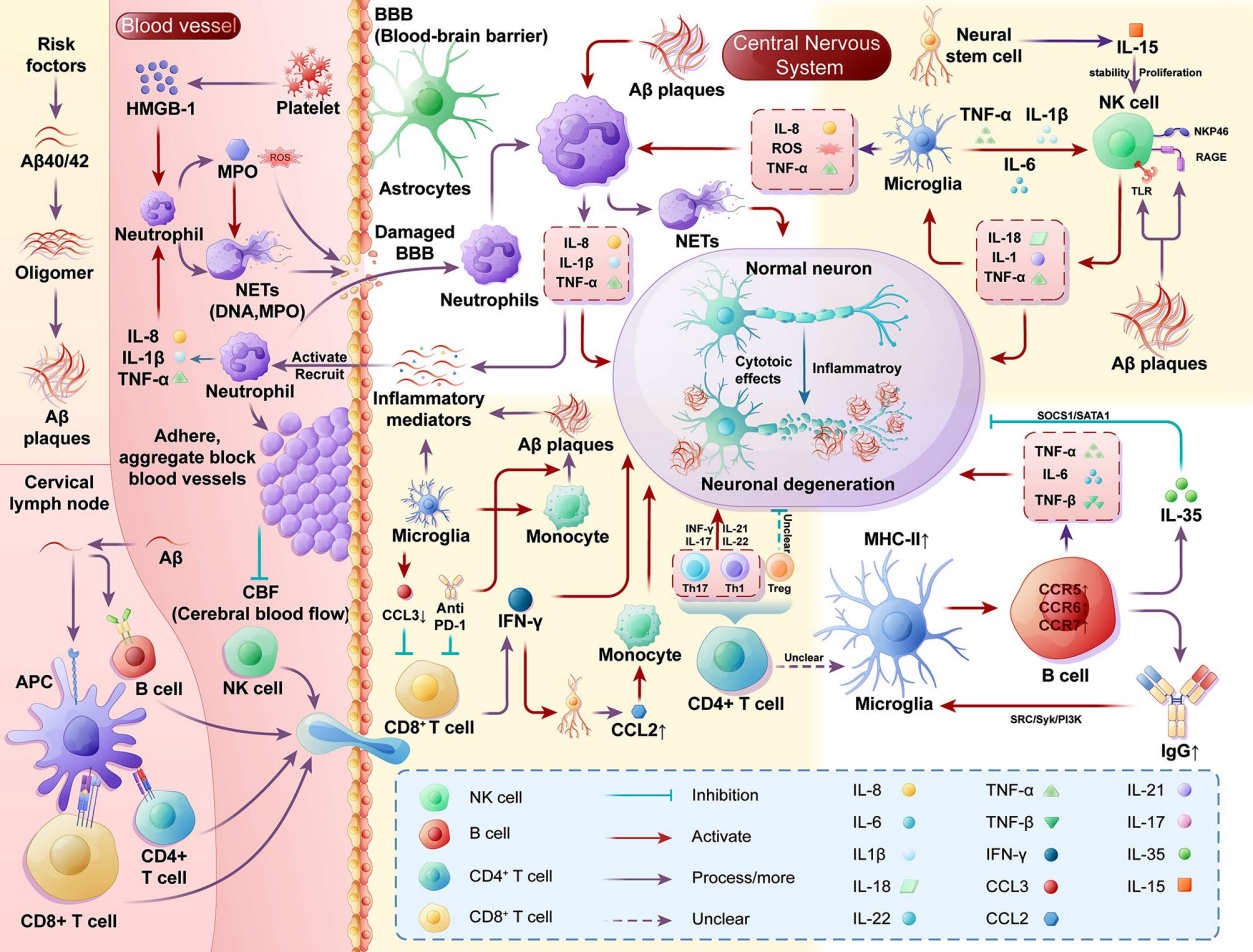
Mechanism of action of neutrophils, T lymphocytes, B lymphocytes and NK cells in Alzheimer’s disease. Neutrophils, T lymphocytes, B lymphocytes, NK cells, and monocytes play crucial roles in the pathological progression of AD. (**A**) β-amyloid (Aβ) reaches the cervical lymph nodes via the bloodstream or lymphatic system, where it is processed and presented by antigen-presenting cells, subsequently activating lymphocytes. These activated lymphocytes then enter the bloodstream and infiltrate brain tissue. (**B**) Neutrophils in the vasculature interact with MPO, HMGB-1, and pro-inflammatory factors, leading to their activation and overexpression of adhesion molecules. This causes them to aggregate and adhere to the vasculature, obstructing blood vessels. Activated neutrophils release neutrophil extracellular traps (NETs) and reactive oxygen species (ROS), which disrupt the blood-brain barrier (BBB). Neutrophils within the brain, influenced by Aβ and microglia, release NETs and pro-inflammatory factors, accelerating the pathological progression of AD. (**C**) After entering the brain, T lymphocytes interact with microglia and Aβ plaques. CD8 + T lymphocytes can directly promote AD pathology, while various CD4 + T lymphocytes modulate neuroinflammation by releasing cytokines, either promoting or inhibiting the process. (**D**) B lymphocytes enhance the phagocytic ability of microglia toward Aβ through the release of IgG and directly delay AD pathology via IL-35. However, they also exacerbate neuroinflammation by releasing pro-inflammatory factors. (**E**) NK cells can be directly activated by Aβ, leading to the release of pro-inflammatory factors. These factors interact with those released by microglia, exacerbating neuroinflammation through a feedback-like mechanism

The pathology of AD is significantly influenced by peripheral immune cells including neutrophils, T lymphocytes, B lymphocytes, and NK cells. Activated neutrophils can accumulate and adhere to vascular walls, potentially obstructing blood flow. They can also enter brain tissue through a compromised BBB, where they worsen the disease by releasing inflammatory cytokines and neutrophil extracellular traps (NETs) [42]. T lymphocytes indirectly contribute to disease progression by affecting glial cell function [43]. B lymphocytes impact the progression of the disease by secreting inflammatory cytokines and antibodies, which then regulate behavior [44–46]. NK cells also contribute to the disease’s progression through interactions with microglia and the release of inflammatory mediators [47, 48]. These complex cellular activities drive the pathogenesis of AD and suggest potential targets for therapeutic intervention. Figure 2 provides a summary of the progress and potential mechanisms of peripheral immune cells and neuroinflammation in AD.

### Neutrophils in AD

#### Activation and migration of neutrophils in AD

The bone marrow constitutes the primary reservoir of neutrophils, which are also present in the bloodstream and the marrow adjacent to the cranial bones within the CNS [49]. Generally, neutrophils circulate within blood vessels and face difficulty traversing the BBB to enter the brain. Consequently, circulating neutrophils lack robust cell adhesion molecules, which prevents their infiltration into the brain parenchyma. In patients with AD, a systemic inflammatory response coupled with a compromised BBB potentially activates neutrophils, facilitating their infiltration into the brain parenchyma [36, 50]. Numerous studies indicate heightened neutrophil activation in the bloodstream of AD patients, evidenced by increased reactive oxygen species (ROS) and NETs detected in their vasculature [51]. This activation mechanism is in stark contrast to that of healthy individuals, where plasma levels of IL-6 and IL-8 are typically lower and not linked to such neutrophil activation. In patients, this activation is orchestrated by cellular factors including C5a, leukotriene B4, platelet-activating factor, and N-Formylmethionyl-leucyl-phenylalanine [51, 52]. Such activation triggers the release of lipid mediators and proteases, amplifying inflammation and attracting additional white blood cells, ultimately worsening tissue damage. Studies using animal models have revealed that mice with cognitive deficits display elevated levels of CD45^+^ and CD18^+^ cells compared to their wild-type counterparts. Furthermore, the detection of Gr-1-positive cells and CAP37 in the cerebral vessels and parenchyma suggests a predominant presence of neutrophils within these regions in AD [42, 53]. Concurrently, a different study employing two-photon microscopy on a mouse AD model observed neutrophil infiltration in the brain parenchyma, with a propensity to migrate towards Aβ plaques [41]. Simultaneously, mature neutrophils express CD177 and LFA-1, enhancing their interaction with CD31 (PECAM-1) and ICAM-1 on endothelial cells and facilitating their infiltration into brain tissue [42, 54]. Nonetheless, this infiltration is typically mild, characterized chiefly by adhesion to endothelial cells. While migrated neutrophils show a preference for Aβ plaques, it is posited that Aβ proteins may not directly attract neutrophils, which necessitates further study [41]. Excessive accumulation of activated neutrophils can disrupt cerebral blood flow and amplify oxidative stress and inflammation, potentially compromising the integrity of the BBB and provoking vascular inflammation [55].

#### Adhesion of neutrophils to vascular endothelium

Neutrophil aggregation has been extensively documented in cerebral blood vessels, particularly adhering to and clustering around areas with Aβ deposition [42]. These activated neutrophils can induce vascular inflammation by disrupting the BBB [50, 55]. A study indicated that neutrophil adhesion to blood vessels could lead to the breakdown of the BBB [55]. It is also implicated in the production of reactive ROS, inflammatory molecules, tissue proteases, and arachidonic acid derivatives that may damage the vascular wall [56]. Upon binding with endothelial cells, the αMβ2 integrin Mac-1 can trigger the release of NETs [57].

Research has shown that neutrophils from 5xFAD mice, which lack the αLβ2 variant of β2 integrin, are unable to enter brain tissue by adhering to endothelial cells [42]. It has been observed that in AD patients, the interaction between LFA-1 and endothelial cell ICAM-1 can cause changes in the cell cytoskeleton and increased permeability of blood vessels [58–60]. In laboratory experiments, it has been found that Aβ proteins can stimulate the expression of endothelial adhesion molecules, including ICAM-1 [61]. As a result, brain vascular endothelial cells in AD patients or AD mouse models show higher levels of ICAM-1 expression, which promotes the adhesion and movement of neutrophils. In mice with early-stage AD who were treated with anti-LFA-1 therapy, there was a decrease in the presence of neutrophils in brain blood vessels and a reduction in their movement out of the vessels, leading to improved cognitive function [42]. Additionally, this study discovered that the treatment also decreased the activation of microglia, a finding that had not been previously reported. This suggests that the interaction between exosmotic neutrophils and microglia can enhance and prolong their activation in the brain. The high activation of neutrophils in AD and their accumulation in blood vessels may be linked to the decreased presence of the endothelial glycocalyx-protein complex [62]. Recent research has identified a new process of neutrophil adhesion in vascular inflammation called Neutrophil DREAM, which enhances neutrophil stickiness during vascular inflammation through both NF-κB-dependent and NF-κB-independent pathways [63]. However, this mechanism has not yet been linked to AD.

#### Neutrophils obstruct cerebral blood flow (CBF)

At the early stages of AD, decreased cerebral blood flow (CBF) has been identified as a prominent sign of onset [64]. Studies conducted using wild-type mice have shown that sustained reduction in CBF to 35% can cause significant cognitive impairments and amplify brain inflammation [65]. Decreased CBF can also disrupt the balance of the CNS by disrupting energy metabolism in the brain [66]. People with AD show increased expression of ICAM-1 in cerebral endothelial cells [42, 67]. This molecule binds to β2 integrins (Mac-1, LFA-1) on neutrophils, enabling them to adhere, crawl, and aggregate in blood vessels, resulting in a reduction in CBF. At the same time, activated platelets also express HMGB1, which has the ability to activate neutrophils, induce cytokine secretion, and increase the expression of VCAM-1 and endothelial cell selectin [68]. After one month of treatment in AD mouse models (3xTg and 5xFAD) with antibodies targeting neutrophils (α-GR-1, α-LFA-1, and α-Ly6G), the study found a significant increase in CBF and continued improvement in cognitive function [42]. It is evident that the decline in CBF observed in AD is not only caused by the accumulation of neutrophils and vasoconstriction, but may also be linked to vascular inflammation and the adhesion of other types of leukocytes. Further research is needed to gain a comprehensive understanding of the issue and its potential treatments. Nevertheless, it is possible that inhibiting neutrophil adhesion to blood vessels could be a viable therapeutic option for AD in the future.

#### The infection-fighting ability of neutrophils in patients with AD

It is generally accepted that AD is a systemic disorder. Neutrophils, which are the first line of defense in the human innate immune system, may be less responsive to external infections in the inflammatory state of AD, thus increasing the risk of infection [69]. Studies have suggested that, although there is a decrease in the peripheral and cerebral anti-infective capabilities of neutrophils in patients with mild cognitive impairment (MCI) and AD in the early stages, they still have the ability to express Toll-like receptor (TLR) receptors [51, 70, 71]. However, there is still debate and conflicting reports regarding this matter. Some studies have indicated that neutrophils in individuals with AD maintain normal phagocytic and degranulation functions [72]. On the other hand, other reports have suggested a decrease in phagocytic function and an increase in degranulation activity among AD patients. Further research is needed to gain a better understanding of these mechanisms and their effects on AD.

#### Neutrophil-associated biomarkers in AD

As key players in inflammation, neutrophils contribute to the synthesis and secretion of biomarkers that are critical for evaluating immune status and diagnosing related diseases. Studies have shown a significant increase in the number and activity of neutrophils in the blood and brain of patients with AD [73]. This increase was positively correlated with disease severity and the rate of progression of mental decline [74]. Therefore, further investigations could potentially unearth new biomarkers for AD, including NETs, myeloperoxidase (MPO), neutrophil gelatinase-associated lipocalin (NGAL), and the neutrophil-to-lymphocyte ratio (NLR). Table 1 lists the relevant studies of MPO, NGAL and NLR in AD in recent years. The changes in these substances in the blood and cerebrospinal fluid are promising markers to reflect the pathological course of AD.

**Table 1:** Overview of neutrophil biomarkers in Alzheimer’s disease Studies

Biomarkers	Sample types	Groups	Numbers	Age (year)	Female/Male	Study types	Methods	Findings (Mean ± SD)	References
NGAL	Plasma (ng/ml)/CSF (pg/ml)	AD	26	79 ± 6	13/13	Retrospective cohort study	ELISA	1494 ± 172, (AD vs. HC, *P* = 0.036); 169 ± 19	[75]
		MCI	28	79 ± 4	14/14			1340 ± 119, (MCI vs. HC, *P* = 0.0027); 152 ± 17	
		HC	26	78 ± 5	13/13			2079 ± 172; 132 ± 13	
	Brain tissue (Entorhinal cortex Hippocampus)	AD	10	78 ± 6	5/5		Western Blot	Significant increase in entorhinal cortex* and hippocampus** (AD vs. HC, *P < 0.05; ** *P* < 0.005)	
		HC	10	82 ± 6	5/5				
	Plasma (ng/ml)	AD	62	72.16 ± 6.35	46/16	Retrospective cohort study	ELISA	191 (AD vs. MCI, *P* = 0.009)	[76]
		MCI	41	69.02 ± 7.77	23/18			296 (MCI vs. HC, *P* = 0.005)	
		HC	38	64.92 ± 5.57	23/15			163	
	Plasma (ng/ml)	AD	19	69.7 ± 6.0	8/11	Retrospective Dual-Center Case-control study	ELISA	65.1 ± 21.0; AUC = 0.766 (AD vs. HC, *P* = 0.002)	[77]
		MCI	27	67.7 ± 9.0	12/15			80.5 ± 21.8; AUC = 0.612 (MCI vs. HC, *P* = 0.155)	
		HC	28	72.1 ± 6.9	19/9			97.6 ± 49.0	
	Serum(ng/ml)	AD	17	78.1 ± 6.4	16/1	Retrospective cohort study	ELISA	(63.8 ± 31.8)/(42.1 ± 22.7), (AD vs. HC, *P* = 0.018)	[78]
		HC	37	71.8 ± 8.1	25/12				
	Plasma (ng/ml)	AD	38	72.8 ± 8.0	18/20	Cross-sectional study	ELISA	(82.0 ± 23.7)/(71.5 ± 20), (AD vs. HC, *P* = 0.007)	[79]
		HC	118	67.4 ± 7.7	73/45				
MPO	Plasma (ng/ml)	AD	28	72.9 ± 9.0	16/12	Prospective cohort study	ELISA	(132.8 ± 114.8)/(55.0 ± 42.6), (AD vs. HC, *P* = 0.002); AUC = 0.745	[80]
		HC	27	67.6 ± 9.7	11/16				
	Brain tissue (Frontal cortex) (MPO^+^ cells/mm^2^)	AD	25	76.4 ± 11.9	12/13	Descriptive Study	Immunohistochemistry	(8.46 ± 0.81)/(5.08 ± 0.62), (AD vs. HC, *P* = 0.003)	[81]
		HC	20	76.3 ± 11.8	9/11				
NLR	Peripheral blood (ratio)	PVFFMC	9760	70.2 (Median)	5750/4010	Retrospective cohort study	Cox regression Restricted cubic spline	AD: when NLR > 5.44, HR = 1.50, [95% *CI*: 1.17–1.93]	[82]
		AD	132	70.83 ± 9.35	69/63	Retrospective cohort study	-	(2.96 ± 2.46)/(1.9 ± 0.66), (AD vs. HC, *P* = 0.005); Cut-point = 1.91, AUC = 0.649, SEN = 65.2%, SPE = 47.4%	[83]
		HC	38	71.09 ± 5.77	13/25				
		AD	56	69.04 ± 9.05	30/23	Retrospective cohort study	Multivariable logistic regression	(2.61 ± 1.04)/(1.88 ± 0.44), (AD vs. HC, *P* < 0.001); OR = 6.27 [95% CI: 3.98–9.82], (*P* = 0.003); Cut-point = 2.35, SEN = 83.05%, SPE = 53.57%;AUC = 0.721, (*P* < 0.001); (2.25 ± 1.01)/(1.88 ± 0.44, (MCI vs. HC, *P* = 0.011); OR = 1.93 [95% CI:1.07–3.47], (*P* = 0.028); Cut-point = 2.34, SEN = 84.38%, SPE = 40.24%; AUC = 0.60, (*P* < 0.03)	[84]
		MCI	57	70.67 ± 9.26	27/30				
		HC	59	68.12 ± 5.81	25/24				
		AD	241	76.53 ± 6.00	165/76	Cross-sectional study	-	(3.21 ± 1.35)/(27.40 ± 4.21), (AD vs. HC, *P* < 0.001); Cut-point = 2.48, SEN = 69.29%, SPE = 79.43%, PPV = 82.3%, NPV = 65.3%; OR = 4.774 [95% CI: 2.821–8.076], (*P* < 0.001)	[85]
		HC	175	71.95 ± 5.40	78/97				
		AD	94	74.2 ± 9.6	54/40	Retrospective cohort study	-	(2.2 ± 1.2)/(1.9 ± 0.7) (AD vs. HC, *P* = 0.09)	[86]
		HC	61	65.7 ± 4.6	30/31				
		MCI	186	73.10 ± 3.29	108/78	Retrospective cohort study	Multivariable logistic regression	(2.39 ± 0.55)/(1.94 ± 0.51) (MCI vs. HC, *P* < 0.001); When NLR ≥ 2.07, OR = 5.993 [95% *CI*: 3.467–10.155, *P* < 0.001], SEN = 73.67%, SPE = 69.3%, PPV = 74.48%, NPV = 68.4%	[87]
		HC	153	71.19 ± 3.32	81/72				

*Abbreviation* AD, Alzheimer’s Disease; AUC, Area Under the Curve; CI, Confidence Interval; HR, Hazard Ratio; HC, Healthy Controls; MCI, Mild Cognitive Impairment; NPV, Negative Predictive Value; OR, Odds Ratio; PPV, Positive Predictive Value; PVFFMC, Patients Visiting for Family Medicine Consultation; SEN, Sensitivity; SPE, Specificity

##### Neutrophil extracellular traps (NETs)

Neutrophils are known to release NETs, which are intricate structures made up of DNA, histones, and a variety of enzymes [88]. Generally, they are known to be a way to trap and stop the movement of bacteria and viruses, thus hindering the transmission of sickness [88]. Excessive NETs or their poor clearance can lead to tissue damage and contribute to various diseases, including viral infections, autoimmune conditions, thrombosis, and cancer progression and angiogenesis [89–92]. When pathogens or inflammatory mediators become activated, neutrophils create ROS, such as superoxide anions. The unraveling of nuclear DNA results in the splitting of the nuclear membrane. The linear DNA combines with histones, basic proteins, and NE in the cytoplasm, forming a fibrous mesh-like structure that is then released into the extracellular space [93, 94]. In animal models of AD, such as 5xFAD and 3xTg-AD mice, neutrophils have been observed infiltrating regions with Aβ deposits and releasing NETs and IL-17. Neutrophils produce IL-17, which can recruit additional neutrophils and directly damage neurons and the BBB [95]. A 2015 study found that autopsies of brains from AD victims showed the presence of neutrophils and NETs in the brain and microvasculature [42]. Previous experiments have indicated that activated endothelial cells release pro-inflammatory cytokines, including tumor necrosis factor (TNF)-α, IL-1β, and IL-8. These cytokines can attract neutrophils and stimulate the release of NETs [96, 97]. Exposing brain endothelial cells to Aβ peptides in a laboratory setting leads to an increase in the expression of cytokine genes, particularly IL-1β. Elevated levels of IL-1β have been observed to activate NETs [98]. The resulting NETs may play a role in AD progression by damaging the BBB and neuronal cells [99, 100].

Studies indicate that platelets significantly contribute to the formation of vascular NETs [68, 101, 102]. Research conducted on mouse models and AD patients has illustrated that when platelets become activated and interact with Aβ deposited in the blood vessels, it can lead to an increase in vascular inflammation [103]. The formation of vascular NETs is likely caused by the interaction between TLR4 and LFA-1 integrin, according to two studies [42, 104]. Activated platelets can release HMGB1, a damage-associated molecular pattern [105]. Neutrophils have receptors for HMGB1, including advanced glycation end products RAGE receptors and TLR4 [104, 106, 107]. HMGB1 activates the production of NETs by neutrophils through interactions with these receptors [102]. Additionally, HMGB1 stimulates neutrophils to produce pro-inflammatory cytokines, which act on endothelial cells and increase the expression of VCAM-1 (Vascular Cell Adhesion Molecule-1) and selectins [42, 108, 109]. Studies have shown that blocking the interaction between RAGE and Aβ can effectively decrease inflammation and neuronal damage. This can be achieved with RAGE antagonists, antibodies, or drugs [102]. Such interventions could prevent Aβ-induced inflammation, minimize neuronal damage, and offer potential therapeutic benefits. However, more research is necessary to assess the efficacy and safety of treatments targeting RAGE.

##### Neutrophil gelatinase-associated lipocalin (NGAL)

The protein known as NGAL, also referred to as lipocalin-2 or siderocalin, is recognized as a potential factor in the progression of AD and depression [77, 110]. In general, NGAL is not highly expressed in peripheral blood and cerebrospinal fluid (CSF) under normal circumstances. However, during AD-associated inflammation, neutrophils release more NGAL, leading to elevated levels of this protein in the bloodstream [111]. Clinical data indicates that individuals with AD and MCI have elevated plasma NGAL levels [73, 76]. Conversely, studies have found no significant difference in NGAL concentrations in the CSF of patients with AD or MCI compared to healthy individuals [112]. An autopsy examination of brain tissue from AD patients showed a marked rise in NGAL levels specifically in the hippocampus [75]. Consequently, while NGAL shows potential as an early biomarker for AD, the variations in its levels in different body fluids imply limitations in its use. The reason for lower NGAL levels in the CSF of AD patients, as one hypothesis suggests, could be due to reduced expression of megalin in the choroid plexus [113].

##### Myeloperoxidase (MPO)

MPO is a protein that is abundantly present in the azurophilic granules of neutrophils. Its essential function includes producing hypochlorous acid and reactive oxygen species, contributing to inflammatory processes [114]. Studies have shown that elevated levels of MPO in the brains of AD patients are linked to increased numbers of neutrophils in the brain vasculature [115]. In the brains of AD patients and the APP/PS1 AD mouse model, MPO co-localizes with neutrophil markers such as CD66B and S100A8 [116]. There is a positive correlation between the increase in peripheral blood MPO levels and Aβ_1–42_ concentration in AD patients [80, 81, 117]. Neutrophil-derived MPO may disrupt the BBB and damage endothelial cells during inflammation [115]. MPO disrupts vascular relaxation by oxidizing endothelium-produced nitric oxide [118]. Neutrophils are known to play a role in the initiation and progression of vascular inflammation, and high levels of MPO produced by these cells can lead to an increase in ROS. This has been linked to the development of AD through oxidative stress. Studies involving APP23 mice that were crossbred with the human MPO gene have shown exacerbated cognitive impairments [119]. However, further research is essential to elucidate the role of neutrophil-derived MPO in the pathogenesis of AD.

##### Neutrophil-to-lymphocyte ratio (NLR)

Recent studies have explored the use of the NLR ratio as a simple and convenient marker of inflammation. Multiple studies have shown that the peripheral blood NLR in patients with AD and MCI is significantly higher than in healthy controls [82, 83, 86, 120, 121]. Multivariate regression analysis indicates NLR as an independent predictor with robust prognostic value for AD [85]. Dong et al. determined that the NLR exhibits high sensitivity and specificity for the diagnosis of AD [84, 85]. The NLR is a composite biomarker that assesses inflammation by analyzing the counts or proportions of neutrophils and lymphocytes. This method is superior to relying solely on the absolute numbers of specific white blood cells because it provides a more nuanced view of the body’s inflammatory status. This method is also superior to relying solely on the absolute numbers of specific white blood cells because it gives a more nuanced view of the body’s inflammatory status.

Recent studies suggest that NETs, NGAL, and MPO could act as potential biomarkers for tracking disease progression and prognosis in AD. Further research is necessary to elucidate the clinical diagnostic and prognostic significance of these biomarkers and to assess their viability as therapeutic targets. Moreover, more investigation is needed to determine the correlations between neutrophil biomarkers, AD-specific biomarkers, and other concurrent pathologies in AD.

### Lymphocytes in AD

#### T lymphocytes in AD

Under normal conditions, lymphocytes cannot cross the BBB into the brain. However, in AD, the permeability of the BBB increases due to elevated levels of adhesion molecules such as VCAM-1 and ICAM-1 [42]. This enhanced permeability facilitates the interaction between the peripheral immune system and the CNS, allowing lymphocyte infiltration into brain tissue. Studies have also discovered that Aβ proteins in the brain can migrate to the cervical lymph nodes through the lymphatic system [122]. Here, antigen-presenting cells capture and present them to T lymphocytes, thus activating the adaptive immune response [123].

Prior studies have shown that AD is characterized by heightened migration of CD4^+^ and CD8^+^ T lymphocytes to the affected regions [124]. Compared to healthy control groups, AD patients show an increased CD4/CD8 ratio and a decreased percentage of CD8 + T lymphocytes in peripheral blood. Increased levels of CD4^+^ and CD8^+^ effector T lymphocytes have been detected in the brain parenchyma of those with AD and MCI [38, 125–127]. However, the precise mechanisms by which T lymphocytes penetrate the BBB and enter the CNS remain elusive. Research has identified distinct subsets of CD4^+^ and CD8^+^ T lymphocytes, including Th1, Th17, and Treg, that are implicated in either promoting or mitigating chronic neuroinflammation in AD [127–131].

##### CD8 + T lymphocytes in AD

Research on CD8^+^ T lymphocytes in the peripheral blood of AD patients has yielded inconsistent results. Earlier studies have indicated lower levels of CD8^+^ T lymphocytes in AD patients, possibly linked to the systemic inflammation characteristic of the disease [121, 132, 133]. However, a recent cohort study found a correlation between clonally amplified CD8 + T T lymphocytes levels in the CSF and cognitive decline in patients with AD [38]. Moreover, infiltration of CD8^+^ T T lymphocytes has been detected in the brains of both AD patients and mouse models of the disease [38, 134, 135]. To enhance our understanding of the role of CD8 + T lymphocytes in AD, Unger et al. conducted a study to investigate the effects of depleting CD8^+^ T lymphocytes using an anti-CD8a antibody in the APP/PS1 mouse model. Their research suggests that brain CD8^+^ T lymphocytes may directly lead to dysfunction in neurons that regulate synaptic plasticity [136].

A recent study investigating peripheral blood T lymphocytes in AD patients found no significant differences in PD-1 expression compared to a healthy control group. However, the same study observed increased PD-L1 expression in CD8^+^ T T lymphocytes, which inversely correlated with cognitive function [137]. While the link between PD-1/PD-L1 expression and cognitive decline requires further investigation, inhibiting PD-1 or PD-L1 has shown promise in enhancing the clearance of brain Aβ plaques by monocyte-derived phagocytic cells and in improving cognition in mouse models [138, 139]. More research is necessary to elucidate the effects of T cell PD-1/PD-L1 expression discrepancies on the pathology of AD. Furthermore, research into the purine receptor P2 × 7R in AD found that mice lacking P2 × 7R (APP/PS1xP2 × 7Rko) exhibited reduced Aβ plaque formation and diminished T cell infiltration in the brain, mediated by lower glial cell CCL3 expression [140]. This effect corresponded with reduced recruitment of CD8^+^ T lymphocytes into the choroid plexus and hippocampus, offering new insights into the interplay between glial cells and T lymphocytes affecting AD pathology [140]. Although blocking P2 × 7R as a means to alleviate Aβ burden in AD has been reported [141–143] it is the inaugural inclusion of first study to include T lymphocytes in such research.

##### CD4^+^ T lymphocytes in AD

CD4^+^ T lymphocytes have garnered significant interest in AD pathology and therapeutic research. Initial comparisons between age-related AD patients and healthy controls indicated a modest elevation in CD4^+^ T lymphocytes [133]. Th17 cells are a subtype of helper T lymphocytes known for secreting cytokines such as IL-17 [144]. Research has identified molecules such as IL-6, TGF-β, and IL-23, along with transcription factors RORγt and NFATc1, as key players in the differentiation of Th17 lymphocytes in patients with AD [127, 145, 146]. Furthermore, AD patients exhibit higher levels of Th17 cell cytokines, including IL-17 and IL-21, compared to individuals with MCI or healthy controls. Although the differences between MCI patients and healthy subjects are marginal, studies show that both MCI and AD patients have significantly increased counts of peripheral blood CD3^+^ CD8^-^ IL-17 A^+^^ +^ Th17 cells than the healthy cohort [126].

Studies have shown that in 3xTg mouse models, IL-17 is able to exacerbate cognitive and synaptic dysfunction in the early stages of Alzheimer’s disease [147]. Preconditioning with an IL-17 antibody through intraventricular injection, or administration via intranasal injection post-induction, can mitigate Aβ_1-42_-induced neurodegeneration and lead to cognitive improvement [148]. Moreover, administration of IL-17 antibody restored acetylcholine-mediated vasodilation, improved hemostasis, and reduced thrombosis [149]. The transfer of Aβ-specific TH17 effector T lymphocytes (Teffs) into the AD mouse model resulted in a significant reduction in postsynaptic protein expression, exacerbating cognitive deficits [131, 150]. Research has shown that Aβ directly modulates the expression of Th17 cytokines, resulting in compromise of the BBB and subsequent infiltration of Th17 cells into the brain parenchyma. This is evidenced by elevated levels of IL-17 and IL-22 within the brain parenchyma [130]. Moreover, IL-17 has been implicated in the induction of cognitive deficits in AD murine models. Cognitive deficits in APP/PS1 mice can be ameliorated by targeting Th17 cell differentiation pathways, such as the IL-12/IL-23 signaling [151]. Intriguingly, patients with AD exhibit elevated levels of IL-23, a cytokine critical for Th17 cell differentiation and activation [152]. However, IL-17 levels do not show a corresponding increase. Nonetheless, heightened expression of RORγt in these patients suggests active involvement of Th17 cells. Furthermore, these cells can produce a considerable amount of IL-21, indicating that there is a subset of Th17 cells whose main function is to secrete IL-21 and promote the development of AD pathology [127]. The precise function of Th17 cells in AD progression is currently unclear. Research has yielded inconsistent outcomes regarding Th17 cell-related cytokine levels and their quantities across various stages of AD. This may be due to the interaction of Th17 cells with other T cell subpopulations. For example, research on Aβ-specific Th1 and Th17 effector cells has demonstrated that their combined presence exacerbates memory deficits and amyloid accumulation in APP/PS1 mice [150]. This contrasts with prior findings indicating that memory could be adversely affected by interferon (IFN)-γ produced by Aβ-specific Th1 cells [131]. Therefore, the researchers suggested that the joint pathological effects of Th1/Th17 effector cells may be activated in culture conditions that contain IFN-γ and IL-12 [150].

##### Regulatory T lymphocytes in AD

Regulatory T lymphocytes (Tregs) are an important part of the immune system because they possess the transcription factors FoxP3, CD25, and CTLA-4, which are responsible for regulating the immune response and promoting immune tolerance [153]. Tregs are known to maintain immune tolerance during disease, but this tolerance may be altered in the context of AD [154]. In AD, effector T lymphocytes respond to misfolded Aβ deposits, undergo clone amplification, and then influence neuroinflammation and AD neuropathology [155]. Regarding the development of Tregs in AD pathology and clinical studies in mouse models, there is a range of reported results, with some studies indicating an increase [156] and others a decrease [157]. Depletion experiments of Foxp3^+^ Tregs [128, 158] and animal research [43] have confirmed the potential neuroprotective effect of Tregs. By constructing Aβ-specific Tregs, some scholars have found that Aβ-specific Tregs can reduce reactive microglia and amyloid deposition through antigen-mediated immunosuppression, thereby improving memory and generating neuroprotective responses [159]. However, D69^+^ Treg cells in the healthy brain can rapidly expand during neuroinflammation, inhibiting astrocyte proliferation through amphiregulin and converting microglia to a pro-inflammatory phenotype through IL-10 secretion [160, 161]. In vitro expansion of clonal Tregs has also been shown to inhibit microglia [162, 163]. The role of Tregs in the progression of AD remains to be further studied. However, due to the powerful anti-inflammatory and immunosuppressive activity of Tregs, adoptive transfer of Treg inducers or polyclonal Tregs can protect against multiple neurodegenerative diseases. Therefore, this immunotherapy targeting Tregs provides a specific immune profile that can improve disease outcomes. The key to developing antigen-specific Treg therapy for AD will be to target Aβ-specific cells that accumulate amyloid-containing parts of the brain. Additionally, Tregs may be able to block immune cells from entering the CNS, reducing the recruitment of immune cells [128, 164].

#### Therapy of AD based on T lymphocytes

Immunotherapy for AD has been a popular field of research, whether through increasing the levels of various cytokines in AD patients [165]. However, the AN1792 Aβ vaccine trial was discontinued due to severe T-lymphocyte meningoencephalitis in 6% of participants. Still, 12 of 59 patients treated with AN1792 developed antibody responses [166, 167]. Subsequent immunotherapies, including the phase 2 bapineuzumab [141] and the phase 3 solanezumab [168, 169], have not improved cognitive impairment in Alzheimer’s patients. Studies have shown that CD4^+^ T lymphocytes expressing Aβ-specific receptors are present in humans [170], and the immune response to Aβ varies according to the MHC genotype [171, 172]. Furthermore, Aβ_33-41_NP has shown efficacy in eliciting specific CD8^+^ T lymphocytes in C57BL/6 and APP/PS1 mice without side effects [173]. Thus, although targeted immunotherapy for AD amyloid shows promise, its safety and effectiveness warrant further investigation. Exploring individual variation, MHC genes, and immune response correlations, along with enhancing treatment safety and efficacy through anchor optimization, is essential.

Studies have aimed at augmenting cytokine levels in Alzheimer’s patients by administering low-dose IL-2 in APP/PS1 mice. This intervention not only boosted the quantity and activity of Tregs but also enhanced amyloid plaque-associated microglia and cognitive performance in mice [128]. Notably, humans have shown good tolerance to low doses of IL-2 [174], indicating its prospective application in clinical settings. Another investigation demonstrated that IL-2 can initiate astrocyte activation and recruitment in the hippocampus, decrease Aβ_42/40_ ratios, lower amyloid plaque load, and enhance synaptic plasticity [175]. Scientists are investigating the use of neurotrophins and T lymphocytes for AD treatment by employing retroviruses to insert brain derived neurotrophic factor (BDNF) into Aβ-specific Th1 cells. This approach successfully prompted the cells to emit BDNF, which decelerates cognitive deterioration in AD sufferers [176]. In a five-year study, mice were observed to investigate the effects of transplantation on amyloid plaques [177]. The results showed that the cells had the capacity to migrate to the plaques, resulting in an increase in synaptic molecules and a decrease in amyloid load. Moreover, increased levels of TrkB and VAMP2 were noted, implying a role for BDNF in synaptic and neural repair. These findings offer fresh perspectives on potential therapy options for AD. Additionally, BACE1 was found to be increased in the plasma of those with AD [178]. In 5xFAD mice treated with Th1-BDNF, there was a noted decrease in BACE1 levels [177]. As a result, BACE1 inhibitors have undergone testing and have been shown to curtail the formation of amyloid plaques [178].

Th1 cells have been found to be effective in cell therapy, and HyeJin Yang et al. further used Aβ peptides to induce the production of Aβ antigen-specific Tregs both in vitro and in vivo, and then conducted adoptive therapy [163]. Following treatment, 3xTg-AD mice exhibited a significant reduction in hippocampal amyloid plaques and lower levels of tau protein phosphorylation [163]. This impact was most notable in the early stages of AD, at 3 months in 3xTg-AD mice. Moreover, the Aβ-specific Treg cells that were transferred survived for more than a week, contributing to the improvement. Research has shown that modulating biological therapy can benefit APP/PS1 and 3XTG-AD mice by activating the STAT4/JAK2/STAT5 pathways, which increases the expression of IFN-γ/IL-10 in CD4^+^ T lymphocytes. This approach has the potential to enhance the brain’s milieu, fostering neurogenesis and cognitive enhancement [178]. These pathways play a crucial role in mediating immune responses, where STAT4 activation promotes the differentiation of Th1 cells, leading to increased production of IFN-γ [179]. JAK2 and STAT5 are involved in signaling that enhances the anti-inflammatory response through the upregulation of IL-10 in CD4^+^ T lymphocytes [180]. The interaction of immune pathways in AD can balance the immune response, reducing neuroinflammation, amyloid plaques, and tau phosphorylation. This may promote neurogenesis and cognitive enhancement. In early AD, boosting Th1 activity is beneficial, while in later stages, enhancing regulatory T cell responses can minimize inflammation and neuronal damage. Immunotherapies offer promise for AD patients by potentially alleviating symptoms and decelerating disease progression. Enhancing the efficacy of T cell therapy requires further research to ascertain optimal cell phenotypes, administration sites, dosages, and delivery methods.

#### B lymphocytes in AD

##### B lymphocytes and neuroinflammation

B lymphocytes, as dedicated APCs, are vital to the functioning of adaptive immunity. Upon stimulation by antigens, B lymphocytes differentiate into plasma cells that secrete antibodies, while concurrently processing antigens for presentation to T lymphocytes. For instance, in AD caused by bacterial infection, B lymphocytes expressing TLR4 on their surface can be stimulated by lipopolysaccharide and induce various neurodegenerative diseases, including AD [181]. This suggests that the immune reaction mediated by B lymphocytes may be involved in the pathological process of AD. The spleen is widely recognized as the primary source of B lymphocytes. However, discrepancies exist in the alterations of B lymphocytes in the spleen and meninges, as observed in the 5×FAD mouse model of AD [182, 183]. One possible explanation is that the meningeal B lymphocytes, which are the main source of B lymphocytes in the brain, originate from the calvaria and undergo negative selection against self-antigens locally [184]. Non-AD transgenic mice expressing APOEε3 and ApoEε4 also display elevated B lymphocyte counts in peripheral blood and pervasive IgG distribution throughout the brain, with the most pronounced effects seen in female APOEε4 transgenic mice [185, 186]. Interestingly, in regions heavily affected by AD, such as the hippocampus, only minimal levels of IgG are detected alongside significant Aβ deposition [187].

Studies using flow cytometry and single-cell RNA sequencing have shown reductions in both absolute counts and relative percentages of B lymphocyte subsets in the blood of AD patients [44, 133]. Furthermore, there is an increase in IgG synthesis and upregulation of the CCR (Chemokine receptor) 5 chemokine receptor, suggesting activation of B lymphocytes in AD [188]. Comparative analyses of healthy elders and AD patients show an elevated level of double-negative (IgD^-^CD27^-^) B lymphocytes [189, 190]. A link has been suggested between early-onset AD (EOAD) and increased circulating double-negative CCR6^+^ B lymphocytes [191]. Additionally, a notable decrease in naive IgD^+^ CD27^-^ B lymphocytes has been observed [192]. RNA sequencing data indicate upregulation of KIR3DL2, PPP2R2B, and QPCT and downregulation of FRAT2 and WWC3 in B lymphocytes, hinting at their possible roles in AD [193–195]. The impact of Ras protein signaling in B lymphocytes in relation to AD remains to be fully elucidated.

CD4^+^ T lymphocytes are critical for B cell activation. Recent research has shown that CD4^+^ T lymphocytes promote MHC-II expression on microglia, enhancing their antigen presentation capabilities [196]. Non-Aβ-specific IgG molecules primarily attach to microglia via the Fc region, activating the Src/Syk/PI3K signaling pathway and boosting microglial phagocytosis of Aβ fibrils [197]. Brain IgG could induce chronic stress-related conditions by activating microglial Fc receptors and causing synaptic disruption and neuroinflammation through the CR3 and FcγR pathways [45, 46]. Changes in TGF-β^+^ microglia can affect B cell function and the secretion of IgG [198]. Depleting B lymphocytes has been shown to lower TGF-β^+^ microglia levels and reduce Trem2, Clec7a, and Itgax expression in the hippocampus, indicating that B lymphocytes may drive chronic microglial dysfunction and amyloid plaque buildup in disease conditions [199]. Tfh cells significantly promote the isotype conversion and antibody secretion of B lymphocytes [96]. The cytokine IL-21, characteristic of Tfh cells, shows a marked elevation in AD patients [200] and has a positive correlation with IgG levels [201]. IL-21 is primarily secreted by Tfh cells in lymph node germinal centers [202] and plays a role in sustaining immunoglobulin class switching and antibody synthesis [203]. This aligns with the elevated Tfh cell populations observed in the spleens of AD mice [201]. Additionally, IL-21 influences Tfh cells to induce Th17 cell differentiation [204], correlating with the rise in Th17 cells seen in AD mice [127]. The activity and differentiation of B lymphocytes are affected by the inflammatory environment. In both aging and AD, B cell subsets and functions have been found to be highly diverse [189, 190]. This implies that B lymphocytes may be involved in the development of AD. The study suggests that B lymphocytes may have a dual role in the neuroinflammation of AD. For example, in APP/PS1 transgenic mice, depletion of B lymphocytes via anti-CD19/B220 antibodies led to heightened hippocampal Aβ deposition and worsened cognition [44]. Additionally, increased B cell numbers, including an IL-35-secreting subset, were observed in the frontal cortex and meninges of 5×FAD mice. It was discovered that IL-35 interacts with neuronal IL-35 receptors, enhancing cognitive function by inhibiting BACE1 transcription through the SOCS1/STAT1 pathway. These results suggest that B lymphocytes could serve as a neuron-specific protective agent and offer a potential avenue for future B cell-focused therapies [197].

In various neurodegenerative diseases, B lymphocytes emit substantial amounts of TNF-α, TNF-β, IL-6, and GM-CSF [205]. Pro-inflammatory factors such as Aβ in AD patients can activate B lymphocytes and transform them into pro-inflammatory B lymphocytes, which then release pro-inflammatory cytokines to promote neuroinflammation and aggravate AD symptoms. Higher expression levels of the pro-inflammatory chemokine receptors CCR5, CCR6, and CCR7 were observed on B lymphocytes of moderate to severe AD [40, 206]. In autoimmune conditions like autoimmune encephalomyelitis and multiple sclerosis, CCR6 influences T cell migration into the cerebrospinal fluid along with selectins and integrins, but this is not observed in AD models [207]. Additionally, CCR7 has proven to be a pro-inflammatory receptor in various chronic inflammatory disease models in both human and mouse studies [208, 209]. These results indicate that the expression of CCR5, CCR6, and CCR7 may be influenced by the pro-inflammatory milieu in AD patients. These cells are similar to DN B lymphocytes associated with AD and the elderly. An elevated presence of DN B lymphocytes is considered contributory to AD development [191].

##### Antibodies in AD

Antibody production is a principal mechanism through which B lymphocytes contribute to adaptive immunity. B lymphocytes also produce autoantibodies that may be implicated in autoimmune diseases or contribute to immune equilibrium. While rare, B lymphocytes can infiltrate the brain via the cerebrospinal fluid and choroid plexus [210]. B lymphocytes can differentiate into plasma cells within cervical lymph nodes in response to stimuli, including Aβ, APOE/Aβ complexes, α-synuclein, and tau proteins [211–213]. In Alzheimer’s patients, the serum contains higher levels of non-specific and Aβ-specific antibodies, which are thought to have neuroprotective properties [214]. These antibodies engage with a range of neurotransmitters and receptors, including N-methyl-D-aspartate (NMDAR), oxidized low-density lipoprotein (oxLDL), and RAGE. Only a fraction of the circulating antibodies can traverse the BBB in healthy mice [215]. In AD, a compromised BBB potentially permits increased antibody ingress into the brain, contributing to pathogenesis through antigen binding. Moreover, heightened IgG levels in the choroid plexus of wild-type mice imply that choroid plexus dysfunction in AD may facilitate brain access for circulating IgG [216].

Both healthy individuals and AD patients can exhibit antibodies against Aβ. In AD patients, these antibodies are thought to interfere with the aggregation and toxicity of Aβ [217]. Studies have discovered that naturally occurring immunity targets several Aβ epitopes, such as the N-terminal A_4_-A_10_ segment, triggering antibody production [218]. Although these antibodies can reduce amyloid plaques, they have not significantly reversed cognitive impairments [219]. Furthermore, antibodies specific to the mid-to C-terminal region of Aβ, beginning at amino acid 28, have been detected (NAbs-Aβ). In healthy individuals, these antibodies can bind to Aβ dimers and trimers, potentially preventing plaque formation and ameliorating cognitive deficits in transgenic mice [217]. Research has produced mixed findings on the presence of anti-Aβ antibodies in both AD patients and healthy subjects. Table 2 (End of manuscript) outlines the diverse outcomes of these experiments. Challenges with enzyme-linked immunosorbent assay -based detection of Aβ antibodies - including immune complex formation and the potential for misinterpretation caused by Aβ monomer adsorption - may result in inconsistent results. Acid treatment to dissociate complexes, ELISpot for measuring soluble Aβ, and specific strategies for addressing antibody complex concerns are possible solutions to these challenges [188].

**Table 2:** Heterogeneity of Aβ antibody levels in body fluids of Alzheimer’s disease patients

Basic information	Type of samples	Research type	Methods	Findings	Country	References
AD patients (*n* = 39), (73.2 ± 8.2) years	Serum	Prospective cohort study	ELISA	Ig anti-Aβ_42_ (Titer): 50 (30 ± 80)/117 (70 ± 200), (AD vs. HC, *P* = 0.02)	United States	[220]
HC (*n* = 39), (77.7 ± 10.8) years				IgG anti-Aβ_42_ (Titer): 295 (180 ± 490)/724 (440 ± 1200), (AD vs. HC, *P* = 0.01)		
AD patients (*n* = 99), (70 ± 8) years, Male/Female(33/63), MMSE (21 ± 5)	Serum	Case-control study	Immunoprecipitation	Anti-Aβ_1−42_ antibody(U): (3.1 ± 3.28)/(5.88 ± 4.54), (AD vs. HC, *P* = 0.001)	Germany	[221]
HC (*n* = 30), (55 ± 9) years, Male/Female (16/14), MMSE (29 ± 1)						
AD patients (*n* = 49); HC (*n* = 46)	CSF	Case-control study	ELISA	Anti-Aβ antibody (Titer) (210.7 ± 23.5)/(303.4 ± 32.78), (AD vs. HC, *P* = 0.02)	United States Germany	[222]
AD patients (*n* = 59), (77 ± 8) years, Male/Female (0.49)	Plasma	Case-control study	ELISA	Reduced anti-CAPS antibodies (AD vs. HC, *P* = 0.018)	United States	[223]
HC (*n* = 59), (70 ± 10) years, Male/Female (0.44)						
AD patients (*n* = 53), (72.3 ± 7.5) years, Male/Female (20/33), MMSE (19.8 ± 6.3)	Serum	Case-control study	ELISA; Dot blot assay	Anti- Aβ_42_, Aβ_1−15_, and Aβ_16−30_ antibodies (Average concentration, µg/ml): (0.59 ± 0.05)/(0.82 ± 0.08), (AD vs. HC, *P* = 0.02)	United States	[224]
HC (*n* = 60), (67.6 ± 5.8) years, Male/Female (39/21), MMSE (28.9 ± 2.6)						
AD patients (Mild, *n* = 8; Moderate, *n* = 9; Severe, *n* = 3), (67.6 ± 6.8) years, MMSE (Mild = 20 ~ 26, Moderate = 14 ~ 19, Severe < 14), Average duration (4.1 ± 2.8) years; HC (*n* = 20), (68.0 ± 7.2) years	Serum	Case-control study	ELISA	Affinity of Aβ antibodies: 0.92 (0.86 ~ 1.09)/1.32 (1.02 ~ 1.41), (AD vs. HC, *P* = 0.03)	China	[225]
AD patients(*n* = 153), (74.89 ± 5.96) years, Male/Female (44/109), MMSE (20.46 ± 6.20)	Serum	Case-control study	ELISA	Anti-Aβ IgG (Titer): 23.3/45.8, (AD vs. HC, *P* < 0.05)	Korea	[226]
HC (*n* = 193), (72.31 ± 5.86) years, Male/Female (81/112), MMSE (27.0 ± 3.08)						
AD patients(*n* = 136), (70.0 ± 10.0) years, Male/Female (100/36), MMSE (17.3 ± 6.9)	Serum	Case-control study	ELISA	Reduced anti-Aβ autoantibody levels (AD vs. HC, *P* < 0.0001)	Korea	[227]
HC (*n* = 210), (70.0 ± 9.8) years, Male/Female (128/82)						
AD patients(*n* = 33), (75.09 ± 8.61) years, Male/Female (19/14), MMSE (14.09 ± 9.23)	Plasma	Case-control study	ELISA	Nearly fourfold (AD vs. HC, *P* = 0.001)	United States	[215]
HC (*n* = 42), (65.67 ± 9.52) years, Male/Female (15/24), MMSE (29.13 ± 1.49)						
AD patients (*n* = 16), (76.9 ± 6.0) years, Male/Female (4/12), MMSE (17.3 ± 3.8)	Serum	Case-control study	ELISA	Aggregated β-amyloid IgG (AD vs. HC, *P* < 0.005); Soluble antibody (AD vs. HC, *P* < 0.05)	United States	[228]
HC (*n* = 31), (72.5 ± 6.4) years, Male/Female (13/18), MMSE (29 ± 1.4)						
AD patients (*n* = 17); HC (*n* = 15)	Serum	Case-control Study	ELISA	Anti-Aβ_25−35_ antibody (Relative units): (161.4 ± 48.8)/(5.6 ± 0.7), (AD vs. HC, *P* < 0.01)	Russia	[229]
AD patients (n1 = 22, n2 = 26), (77.0 ± 7.0)/(79.0 ± 5.0) years, Duration (n1 < 5 years, n2 > 10 years)	Serum	Case-control study	ELISA Dot blot hybridization	Elevated anti-Aβ25–35 oligomer (AD vs. HC, *P* < 0.05)	Russia	[230]
HC (*n* = 28), (75.0 ± 4.0) years, MMSE (27)						
AD patients (*n* = 54), (52–91) years	Serum	Comparative study	ELISA	Significantly higher optical density of Aβ antibodies (AD vs. HC, *P* < 0.001)	United States	[231]
HC (*n* = 49), (64–90) years						
AD patients (*n* = 82), (76.0 ± 7.11) years, Male/Female (22/64)	Plasma	Prospective cohort study	ELISA	Anti-aβ antibody (Average titer): (16.07 ± 17.5)/(16.96 ± 41.5), (AD vs. HC, *P* < 0.05)	United States	[232]
HC (*n* = 271), (75.8 ± 5.9) years, Male/Female (87/184)						
AD patients (*n* = 36), (76.5) years, Male/Female (13/23), Average MMSE (17)	Serum	Case-control study	ELISA	Log10 IgG to Ab40: (2.83 ± 0.29)/(2.78 ± 0.19), (AD vs. HC, *P* = 0.19) Log10 IgG to Aβ42: (2.95 ± 0.39)/(2.83 ± 0.19), (AD vs. HC, *P* = 0.056)	France	[233]
HC (*n* = 34), (72.0) years, Male/Female (7/27), Average MMSE (29)						
AD patients (*n* = 113), (75) years, Male/Female (32/81), MMSE (14.9 ± 6.7)	Plasma	Case-control study	Tissue amyloid plaque immunoreactivity (TAPIR)	High incidence of TAPIR: 45.1%/41.3%, (AD vs. HC, *P* = 0.77)	Japan	[234]
HC (*n* = 155), (76) years, Male/Female (59/96), MMSE (29.7 ± 0.4)						

*Abbreviation* AD, Alzheimer’s Disease; CAPS, Cross-linked beta-amyloid protein species; CSF, Cerebrospinal fluid; ELISA, Enzyme-linked immunosorbent assay; HC, Healthy controls; MMSE, Mini-Mental State Examination; TAPIR, Tissue amyloid plaque immunoreactivity

Damage to neurons and synapses in AD results in the release of tau proteins, neurofilaments, and microtubule-associated proteins, which can stimulate B lymphocytes to produce specific antibodies [235]. Research indicates that immunoglobulins can bind to tau and cytoskeletal proteins, potentially modifying their structure and activity, and contributing to the pathology of neurodegenerative diseases [236]. In TAU58/2 mice, a deficiency of B lymphocytes has been linked to the exacerbation of cognitive deficits. Conversely, long-term B cell depletion via anti-CD20 antibodies did not lead to worsening of cognitive deficits [237].

##### B lymphocytes dependent therapy in AD

Although the Aβ-IgG complex in cerebrospinal fluid has been linked to cognitive performance in patients with AD, this does not necessarily mean that it has a positive effect on improving cognitive impairment [238]. Indeed, less than 0.0017% of administered immunoglobulin reaches the brain post-intravenous injection, contributing to its limited effectiveness in treating AD [239, 240]. Consequently, this therapeutic approach may be suboptimal. NMDARs, a class of ionotropic glutamate receptors, play crucial roles in synaptic activity, memory development, and controlling neuronal damage due to overstimulation [241]. Dysfunctional NMDARs in AD hinder neuronal communication, which is a reason why memantine, an NMDAR antagonist, is used to treat moderate to severe AD [242]. Agents like memantine, non-competitive NMDAR antagonists, can suppress B cell response to BCR and TLR4 signals, leading to reduced calcium entry and signaling, decreased levels of IgM and IgG, and lower B cell mobility and viability. Conversely, ifenprodil, another NMDAR antagonist employed in depression therapy, can instigate the BCR/cd40 pathway, stimulating the production of the anti-inflammatory cytokine IL-10 by B lymphocytes. The divergent effects likely result from differences in drug timing and dosage, necessitating additional research to uncover the underlying molecular processes [243].

### NK cells in AD

Natural killer (NK) cells play a crucial role in innate immunity and are divided into two subsets: CD56bright and CD56dim. The CD56bright subset produces cytokines and chemokines to regulate inflammation and the immune response, while the CD56dim subset releases cytotoxic molecules to destroy tumor and infected cells [244]. The risk of AD increases with age, and studies have shown an increase in NKp46^+^ cells in the dentate gyrus of elderly individuals and aging mice [39]. Aging neural progenitor cells release factors that attract NK cells to aging areas and upregulate genes associated with immune regulation, cellular senescence, and DNA damage response. In both human AD patients and 3xTG-AD mice [47, 245–248]. NK cells have been observed to enter the brain’s circulatory system, primarily located in the choroid plexus, pial meninges, and surrounding vasculature with limited penetration into the brain parenchyma [47].

NK cells can be categorized into different subgroups, such as NK1AD cells (Thy1-CD7-/low) that have high levels of cytotoxic molecules (Ctsc, Ctsd), pro-inflammatory chemokines (CCL3, 4), adhesion molecules (ICAM-1), and lymphocyte activation molecules (Nfatc1, Tbx21, Nfkbia, Il12rb, and Klra9). Studies have shown that both NK1a cells, which have low levels of cytotoxic molecules, and NK1b cells, which have high levels of cytotoxic molecules, exhibit increased activity [47]. In mice, treatment with anti-NK_1.1_ improved cognitive function and reduced the expression of genes related to microglial proliferation (Mki67, Cdc42) and pro-inflammatory cytokines (TNF, IL-1, IL-18), while maintaining the microglia’s ability to clear Aβ [249]. In AD, inflammatory molecules such as Aβ and IL-18 activate NK cells through receptors like RAGE and TLR [250, 251]. This activation leads to increased secretion of cytokines, including IL-1, IL-18, and TNF, creating a feedback loop that worsens neuroinflammation and cognitive decline. In comparison to healthy individuals, NK cells in AD patients show heightened responsiveness to IL-2 and IFN-β, with IL-2 stimulating the production of IFN-γ and TNF-α [245]. Analysis of NK cell transcriptomes in AD patients reveals decreased levels of cytotoxic (NK0) and adaptive (NK2) subgroups in the blood, but an increased presence in the brain [252]. Bioinformatics analysis identified 17 transcription factors shared between NK cells in the blood and AD brain tissue, with 13 showing increased expression [48].

Research has shown a connection between the activation of STAT3 and the infiltration of immune cells in tumors. Inhibiting STAT3 has been linked to a decrease in NK cell infiltration and IFN-γ levels. These findings suggest that STAT3 may play a role in NK cell activation and brain infiltration in AD, but further research is needed to confirm this [253]. Analysis of NK cells in the brains of 3XTg-AD mice revealed more activated clusters, indicating active NK cell infiltration in the Alzheimer’s brain [48]. Treating 3XTg-AD mice with anti-NK_1.1_ resulted in fewer EdU^+^ NeuN^+^ cells in certain brain regions, suggesting enhanced neurogenesis 47.Serotonin, a neurotransmitter that plays a role in emotions, sleep, learning, and memory, has been shown to moderately improve cognitive function in Alzheimer’s patients. AD patients have been found to have increased expression of the 5-HT2C receptor on NK cells, which may be related to the decreased serotonin levels often seen in these patients, who commonly experience depression and anxiety [254, 255]. Despite evidence showing serotonin’s dose-dependent inhibition of NK cell cytotoxicity, more research is necessary to investigate the impact of this cytotoxicity on AD progression.

Recent studies have shown that neural stem cells play a significant role in regulating the activity of NK cells in the brain during neuroinflammation [256]. NSCs release high levels of IL-15, which is crucial for the proliferation and function of NK cells. This interaction between IL-15 and IL-15Rα is essential for the survival of NK cells [257]. However, more research is needed to fully understand the role of NK cells in AD and their impact on neuroinflammation. Further investigation into their interaction with glial cells and their potential role in nerve regeneration could provide valuable insights and lead to the development of targeted therapies for AD.

### Monocytes

#### Recruitment and migration of monocytes

Monocytes, a crucial component of the peripheral mononuclear phagocyte system, have been found to participate in the clearance of tau protein in AD [258]. However, current studies are limited to the clearance of tau protein in peripheral blood, which can indirectly alleviate tau protein phosphorylation and neuronal damage in the brain [259]. Similar to tau, the diffusion of Aβ from the brain to the peripheral blood reaches 40-60% [260]. In AD mouse models, this portion of Aβ is solely cleared by the mononuclear phagocyte system [261]. Therefore, studies suggest that the migration of monocytes to the brain has neuroprotective effects on the CNS [262]. The process of monocyte infiltration into the brain is complex, generally involving the initial penetration of the endothelial monolayer and translocation across the basement membrane. This involves briefly residing on the basement membrane after crossing the endothelial layer and finally relies on the proteolytic activity of matrix metalloproteinases MMP-2 and MMP-9 to traverse the basement membrane and astrocytic end-feet [263]. Under physiological conditions, microglia can self-renew [264]; however, in AD, this regenerative capacity is impaired [265], potentially inducing the recruitment of peripheral monocytes to the brain to compensate for the damaged microglia [266]. These recruited monocytes are predominantly CCR2^+^ monocytes. However, the number of CCR2^+^ monocytes in the peripheral blood and bone marrow of AD patients is decreased [267], suggesting that this spontaneous recruitment is insufficient to sustain such protective functions. Additionally, whether these recruited monocytes can differentiate into microglia within the AD brain remains unclear [268], and these two cell types are difficult to distinguish based on morphology and marker expression. An in vitro study induced human peripheral blood monocytes into monocyte-derived induced microglia-like cells (MDMi) via Aβ oligomers, and observed an increase in phagocytic index and the upregulation of 20 genes. Interestingly, the gene expression profile of MDMi differed from that of microglia observed in the later stages of AD pathology [269]. Although this finding supports the notion that monocytes may perform functions distinct from resident microglia after migrating to the brain, the artificial nature of the in vitro environment prevents definitive conclusions.

CCL2 (MCP-1) is a chemokine for innate immune cells, and its secretion is increased in the innate immune cells of AD patients [270]. CCL2 plays a crucial role in the infiltration of monocytes through the BBB into the brain [271]. Previous studies have demonstrated that Aβ_42_ can promote the expression of CCL2 in monocytes [272], thereby facilitating their infiltration into the inflamed brain, leading to localized inflammation. Additionally, another study found that MDP (Muramyl dipeptide, a minimal bioactive peptidoglycan motif from most Gram-negative and Gram-positive bacteria) could also enhance CCL2 expression in monocytes. Interestingly, the levels of downstream inflammatory mediator NF-κB did not show significant changes, suggesting that MDP can increase CCL2 expression and promote monocyte recruitment to brain vasculature independently of pro-inflammatory responses [273]. Additionally, in tau pathology mouse models, targeting the PD-1/PD-L1 pathway has been shown to enhance the recruitment of monocyte-derived macrophages into the brain parenchyma. This recruitment decreases inflammation and reduces cognitive deficits in the brain [139].

#### The phagocytosis of monocytes decreased

AD predominantly affects elderly individuals. As aging progresses and the disease advances, the energy metabolism status of various immune cells, including monocytes and microglia, becomes impaired. Their glucose metabolism and oxidative phosphorylation levels notably decrease [274], which significantly impacts the functions of these immune cells. The use of an EP2 receptor antagonist (PF-04418948) to inhibit the PGE2-EP2 pathway can restore the energy metabolism of aged monocyte-derived macrophages [275], reverse cellular aging, and enhance their phagocytic functions. This treatment reduces Aβ burden and tau phosphorylation levels, while simultaneously decreasing IL-1β and increasing IL-10 levels in the brain, thereby improving cognitive impairments in mice [276]. In elderly and AD populations, significant changes in peripheral blood serum components include elevated levels of inflammatory markers (c-reactive protein, IL-6, TNF-α) [277]. This chronic systemic inflammatory response is not solely derived from brain pathology but it is also closely associated with the involvement of aged peripheral immune cells. Under the dual influence of decreased energy metabolism and a pro-inflammatory environment, monocytes not only fail to phagocytize Aβ but also release pro-inflammatory factors that affect the function of other cells [278].

In addition to energy metabolism disorders and pro-inflammatory environments, the pathogenesis is also related to the expression of cell surface membrane proteins. The CD33 gene has been previously identified as a risk factor for AD, and the CD33 protein it encodes is a sialic acid-binding immunoglobulin-like lectin that regulates innate immunity. Previous research indicates that CD33 expression is increased on microglia in the AD brain, and it inhibits microglial uptake of Aβ [279]. Similarly, another study found that inhibition of CD33 expression in mice leads to enhanced uptake of Aβ by monocyte-derived macrophages and microglia, thereby reducing Aβ accumulation [279]. Additionally, studies have shown that TLR2 expression is decreased in AD. This receptor forms a complex with CD14 to recognize and uptake Aβ [280]. PSK (Polysaccharide Krestin), a cancer treatment drug, can activate anti-tumor immune responses by promoting the maturation of dendritic cells [281]. In AD mouse models, PSK can increase peripheral Aβ uptake and intracellular metabolism by monocytes through the TLR2-mediated pathway, thereby alleviating cognitive impairments in mice [282]. Moreover, PSK not only targets Aβ but also inhibits the excessive phosphorylation of tau proteins, thus reducing neuroinflammation and neuronal damage [282]. This indicates that enhancing and activating the TLR2 pathway can improve monocyte function and intervene in the pathogenesis of AD.

Erythropoietin (EPO) has shown immunomodulatory potential and beneficial effects in the treatment of AD. However, its clinical application is hindered by the risk of thrombosis due to erythropoiesis induction. EPO can activate the innate repair receptor (IRR), which comprises the EPOR and β common receptor (βCR), providing neuroprotection in AD mouse models without eliciting erythropoietic effects [283]. ARA290, a peptide derived from EPO, also activates the IRR and regulates immune function without inducing erythropoiesis [284]. Early use of ARA290 in APP/PS1 mice reduces Aβ pathology in the brain and alleviates cognitive impairments without altering microglial reactivity. ARA290 increases the number of circulating monocytes, and the depletion of these monocytes negates the cognitive benefits provided by ARA290. This suggests that monocytes can potentially delay AD progression by phagocytizing Aβ. However, this therapeutic effect is limited in late-stage AD models [285].

#### Monocytes are associated with cerebrovascular amyloidosis

As previously mentioned, anti-amyloid immunotherapy has been tested in multiple late-stage clinical trials. Although patients receiving these antibody treatments have shown improvements in soluble biomarkers, amyloid pathology, and cognitive function, there have been occurrences of amyloid-related brain edema or microhemorrhage, which pose significant risks for a therapeutic drug [286, 287]. The occurrence of these side effects is not only related to T lymphocytes but may also involve monocytes.

During the normal vascular development of the CNS, perivascular macrophages directly interact with developing blood vessels, influencing various stages of the angiogenesis process [288] and aiding in the clearance of vascular Aβ deposits in AD mouse models [289]. In Cerebral amyloid angiopathy associated with AD, Aβ deposits along the drainage pathways around cerebral blood vessels between the endothelium and the basement membrane. Besides the direct disruption of the vascular wall by Aβ, which increases BBB permeability [279, 284], it also promotes the expression of pro-inflammatory cytokines, exacerbating vascular inflammation and facilitating monocyte-macrophage infiltration. For instance, the use of Bapineuzumab and mouse monoclonal antibody equivalent 3D6 in anti-Aβ immunotherapy can reduce total Aβ levels but also induces significant inflammatory responses, enhances vascular permeability, and compromises vascular integrity [290]. Subsequently, Xavier et al. discovered that CD169^+^ perivascular mononuclear macrophages could be activated by the 3D6-Aβ antibody complexes, which is associated with increased plasma protein leakage and microhemorrhages in PDAPP mice [291]. Deposited amyloid can stimulate the release of reactive oxygen species by monocytes, leading to increased oxidative stress in cerebral vessels [292]. These findings underscore the critical involvement of perivascular mononuclear macrophages in Cerebral amyloid angiopathy-mediated vascular permeability and microhemorrhages associated with amyloid immunotherapy, potentially through amplification of the local inflammatory milieu and remodeling of the extracellular environment around vascular amyloid deposits [293].

## Conclusion

Research on the interactions of immune cells, such as neutrophils, T lymphocytes, B lymphocytes, NK cells, and Monocytes is a growing area of interest in AD. The specific ways in which these cells contribute to the development of AD are not fully understood. We have summarized the known or potential roles of peripheral immune cells in AD in Table 3. Studying how neutrophils migrate could provide new insights into their role in AD. Further research is needed to understand the regulatory effects of T lymphocytes, particularly CD8^+^ cells, and their impact on AD. The mechanisms by which CD4^+^ T lymphocytes influence MHC-II expression in microglia and their effects on AD require more investigation. The role of Ras signaling in B lymphocytes in AD progression is not well understood and needs more study. Additionally, the activation and infiltration of NK cells in the AD brain through STAT3 signaling needs to be further explored to clarify their role in the disease. The biggest direction for future monocytes in AD research is to further investigate their recruitment, migration and phagocytosis functions to enhance their ability to clear tau and Aβ proteins and reduce the side effects of anti-Aβ immunotherapy. Addressing these knowledge gaps could lead to the discovery of new diagnostic markers, therapeutic targets, and preventive strategies for AD.

**Table 3:** The contribution of peripheral immune cells to the pathological development of AD at different stages

Peripheral immune cells	Models	Stages	Effects	Potential mechanism	Reference
Neutrophils	5xFAD and 3xTg-AD mice	Early stage	Promote inflammation.	Neutrophils promote AD-like pathology and cognitive decline via the LFA-1 integrin.	[42]
	APP/PS1 and 5xFAD mice	Not mentioned	Reduces cortical blood flow and impairs memory function.	Antibodies against the neutrophil marker Ly6G reduced the number of stalled capillaries, resulting in an immediate increase in CBF and rapid improvement in spatial and working memory tasks.	[55]
Th17	Rat	Not mentioned	Promote inflammation.	Involvement in neuroinflammation and neurodegeneration through pro-inflammatory cytokines and the Fas/FasL apoptosis pathway.	[130]
CD8^+^ T lymphocytes	APP/PS1 transgenic mice	Not mentioned	Infiltrate the brains of individuals with Alzheimer’s disease and regulate the expression of genes related to neurons and synapses.	Infiltrate the aged and AD brain, and the brain’s CD8^+^ T-cells may directly contribute to neuronal dysfunction by modulating synaptic plasticity.	[136]
γδ T lymphocytes	3xTg-AD mice	Not mentioned	The accumulation of IL-17 in the brain and meninges leads to cognitive and synaptic dysfunction.	This may be related to IL-17 and a finely regulated balance of “inflammatory” cytokines derived from the meningeal immune system.	[147]
Tregs	APP/PS1 mice	Not mentioned	Amyloid-β-specific regulatory T cells attenuate the pathobiology of Alzheimer’s disease.	Related to the adoptive transfer of TCRAβ-Tregs, there was sustained immune suppression, reduced microglial reaction, and decreased amyloid loads.	[159]
			Delay disease progression in Alzheimer-like pathology.	It may be related to slowing disease progression and modulating the microglial response to amyloid-β deposition.	[128]
B lymphocytes	Rag-5xfAD mice	Not mentioned	The adaptive immune system restrains Alzheimer’s disease pathogenesis by modulating microglial function.	This may be related to IgG inducing Aβ phagocytosis via a Src/Syk/PI3K signal transduction pathway.	[197]
	3xTg-AD mice	Not mentioned	Increased expression of CCR6 in the brain and peripheral immune organs of both pre-symptomatic and symptomatic 3xTg-AD mice.	Unknown.	[206]
NK cells	3xTg-AD mice	Not mentioned	Promote inflammation.	This may be related to the pro-inflammatory function of NK cells.	[47]
	AD patients	AD	Significant negative correlations were found among the spontaneous release of IFN-gamma and TNF-alpha from NK cells and the decrease in the score of cognitive function (MMSE) in patients with DAT.	This may be related to the control of NK cell cytotoxicity and the release of NK-derived cytokines in AD, which could be involved in the neuroinflammatory mechanism related to disease progression.	[245]
Monocytes	Tg2576 mice	Not mentioned	Promote brain perivascular monocytes initiate the neurovascular dysfunction of Alzheimer’s Aβ peptides.	Through the innate immune receptor CD36, which activates a Nox2-containing NADPH oxidase, leading to cerebrovascular oxidative stress.	[292]

## Data Availability

No datasets were generated or analysed during the current study.

## Abbreviations

Aβ: Amyloid-beta
AD: Alzheimer’s disease
APCs: Antigen-presenting cells
BBB: Blood-brain barrier
BDNF: Brain derived neurotrophic factor
CBF: Cerebral blood flow
CCR: Chemokine receptor
CNS: Central nervous system
IFN: Interferon
IL: Interleukin
MCI: Mild cognitive impairment
MPO: Myeloperoxidase
NE: Neutrophil elastase
NETs: Neutrophil extracellular traps
NFTs: Neurofibrillary tangles
NLR: Neutrophil-to-lymphocyte ratio
NGAL: Neutrophil gelatinase-associated lipocalin
TNF: Tumor necrosis factor

## References

[1] Nandi A, Counts N, Chen S, Seligman B, Tortorice D, Vigo D (2022). Global and regional projections of the economic burden of Alzheimer’s disease and related dementias from 2019 to 2050: a value of statistical life approach. EClinicalMedicine.

[2] 2022 Alzheimer’s disease facts and figures. Alzheimer’s & dementia: the journal of the Alzheimer’s Association. 2022; 18(4): 700 – 89. 10.1002/alz.12638 35289055

[3] Scheltens P, De Strooper B, Kivipelto M, Holstege H, Chételat G, Teunissen CE (2021). Alzheimer’s disease. Lancet (London England).

[4] Yamazaki Y, Zhao N, Caulfield TR, Liu CC, Bu G (2019). Apolipoprotein E and Alzheimer disease: pathobiology and targeting strategies. Nat Reviews Neurol.

[5] Poirier J (2003). Apolipoprotein E and cholesterol metabolism in the pathogenesis and treatment of Alzheimer’s disease. Trends Mol Med.

[6] DeMattos RB, Cirrito JR, Parsadanian M, May PC, O’Dell MA, Taylor JW (2004). ApoE and clusterin cooperatively suppress abeta levels and deposition: evidence that ApoE regulates extracellular Abeta metabolism in vivo. Neuron.

[7] Ferrari-Souza JP, Lussier FZ, Leffa DT, Therriault J, Tissot C, Bellaver B (2023). APOEε4 associates with microglial activation independently of Aβ plaques and tau tangles. Sci Adv.

[8] Lane-Donovan C, Herz J, ApoE (2017). ApoE receptors, and the synapse in Alzheimer’s Disease. Trends Endocrinol Metab.

[9] Sivanandam TM, Thakur MK (2012). Traumatic brain injury: a risk factor for Alzheimer’s disease. Neurosci Biobehav Rev.

[10] Tosto G, Bird TD, Bennett DA, Boeve BF, Brickman AM, Cruchaga C (2016). The Role of Cardiovascular Risk factors and stroke in familial Alzheimer Disease. JAMA Neurol.

[11] Dong Z, Xu M, Sun X, Wang X (2023). Mendelian randomization and transcriptomic analysis reveal an inverse causal relationship between Alzheimer’s disease and cancer. J Translational Med.

[12] De Chiara G, Piacentini R, Fabiani M, Mastrodonato A, Marcocci ME, Limongi D (2019). Recurrent herpes simplex virus-1 infection induces hallmarks of neurodegeneration and cognitive deficits in mice. PLoS Pathog.

[13] Kivimäki M, Singh-Manoux A, Pentti J, Sabia S, Nyberg ST, Alfredsson L (2019). Physical inactivity, cardiometabolic disease, and risk of dementia: an individual-participant meta-analysis. BMJ (Clinical Res ed).

[14] Lennon MJ, Koncz R, Sachdev PS (2021). Hypertension and Alzheimer’s disease: is the picture any clearer?. Curr Opin Psychiatry.

[15] Baril AA, Carrier J, Lafrenière A, Warby S, Poirier J, Osorio RS (2018). Biomarkers of dementia in obstructive sleep apnea. Sleep Med Rev.

[16] Masters CL, Selkoe DJ (2012). Biochemistry of amyloid β-protein and amyloid deposits in Alzheimer disease. Cold Spring Harbor Perspect Med.

[17] Bernstein SL, Dupuis NF, Lazo ND, Wyttenbach T, Condron MM, Bitan G (2009). Amyloid-β protein oligomerization and the importance of tetramers and dodecamers in the aetiology of Alzheimer’s disease. Nat Chem.

[18] Shea D, Hsu CC, Bi TM, Paranjapye N, Childers MC, Cochran J (2019). α-Sheet secondary structure in amyloid β-peptide drives aggregation and toxicity in Alzheimer’s disease. Proc Natl Acad Sci USA.

[19] Santos AN, Ewers M, Minthon L, Simm A, Silber RE, Blennow K (2012). Amyloid-β oligomers in cerebrospinal fluid are associated with cognitive decline in patients with Alzheimer’s disease. J Alzheimer’s Disease: JAD.

[20] Arber C, Lovejoy C, Harris L, Willumsen N, Alatza A, Casey JM (2021). Familial Alzheimer’s disease mutations in PSEN1 lead to premature human stem cell neurogenesis. Cell Rep.

[21] Morice R (1986). Beyond language–speculations on the prefrontal cortex and schizophrenia. Aust N Z J Psychiatry.

[22] van der Lee SJ, Wolters FJ, Ikram MK, Hofman A, Ikram MA, Amin N (2018). The effect of APOE and other common genetic variants on the onset of Alzheimer’s disease and dementia: a community-based cohort study. Lancet Neurol.

[23] Livingston G, Huntley J, Sommerlad A, Ames D, Ballard C, Banerjee S (2020). Dementia prevention, intervention, and care: 2020 report of the Lancet Commission. Lancet (London England).

[24] Frisoni GB, Altomare D, Thal DR, Ribaldi F, van der Kant R, Ossenkoppele R (2022). The probabilistic model of Alzheimer disease: the amyloid hypothesis revised. Nat Rev Neurosci.

[25] Drubin DG, Kirschner MW (1986). Tau protein function in living cells. J Cell Biol.

[26] Kobayashi S, Tanaka T, Soeda Y, Almeida OFX, Takashima A (2017). Local somatodendritic translation and hyperphosphorylation of tau protein triggered by AMPA and NMDA receptor stimulation. EBioMedicine.

[27] Weller RO, Preston SD, Subash M, Carare RO (2009). Cerebral amyloid angiopathy in the aetiology and immunotherapy of Alzheimer disease. Alzheimers Res Ther.

[28] Yang T, Zhu Z, Yin E, Wang Y, Zhang C, Yuan H (2019). Alleviation of symptoms of Alzheimer’s disease by diminishing Aβ neurotoxicity and neuroinflammation. Chem Sci.

[29] Selkoe DJ (2002). Alzheimer’s disease is a synaptic failure. Sci (New York NY).

[30] Langworth-Green C, Patel S, Jaunmuktane Z, Jabbari E, Morris H, Thom M (2023). Chronic effects of inflammation on tauopathies. Lancet Neurol.

[31] Leng F, Edison P (2021). Neuroinflammation and microglial activation in Alzheimer disease: where do we go from here?. Nat Reviews Neurol.

[32] Yasojima K, Schwab C, McGeer EG, McGeer PL (1999). Up-regulated production and activation of the complement system in Alzheimer’s disease brain. Am J Pathol.

[33] Liang T, Zhang Y, Wu S, Chen Q, Wang L (2022). The role of NLRP3 inflammasome in Alzheimer’s Disease and potential therapeutic targets. Front Pharmacol.

[34] Fonseca MI, Zhou J, Botto M, Tenner AJ (2004). Absence of C1q leads to less neuropathology in transgenic mouse models of Alzheimer’s disease. J Neuroscience: Official J Soc Neurosci.

[35] Liu L, Chan C (2014). The role of inflammasome in Alzheimer’s disease. Ageing Res Rev.

[36] Bowman GL, Kaye JA, Moore M, Waichunas D, Carlson NE, Quinn JF (2007). Blood-brain barrier impairment in Alzheimer disease: stability and functional significance. Neurology.

[37] Reale M, Iarlori C, Feliciani C, Gambi D (2008). Peripheral chemokine receptors, their ligands, cytokines and Alzheimer’s disease. J Alzheimer’s Disease: JAD.

[38] Gate D, Saligrama N, Leventhal O, Yang AC, Unger MS, Middeldorp J (2020). Clonally expanded CD8 T cells patrol the cerebrospinal fluid in Alzheimer’s disease. Nature.

[39] Jin WN, Shi K, He W, Sun JH, Van Kaer L, Shi FD (2021). Neuroblast senescence in the aged brain augments natural killer cell cytotoxicity leading to impaired neurogenesis and cognition. Nat Neurosci.

[40] Subramanian S, Ayala P, Wadsworth TL, Harris CJ, Vandenbark AA, Quinn JF (2010). CCR6: a biomarker for Alzheimer’s-like disease in a triple transgenic mouse model. J Alzheimer’s Disease: JAD.

[41] Baik SH, Cha MY, Hyun YM, Cho H, Hamza B, Kim DK (2014). Migration of neutrophils targeting amyloid plaques in Alzheimer’s disease mouse model. Neurobiol Aging.

[42] Zenaro E, Pietronigro E, Della Bianca V, Piacentino G, Marongiu L, Budui S (2015). Neutrophils promote Alzheimer’s disease-like pathology and cognitive decline via LFA-1 integrin. Nat Med.

[43] Ito M, Komai K, Mise-Omata S, Iizuka-Koga M, Noguchi Y, Kondo T (2019). Brain regulatory T cells suppress astrogliosis and potentiate neurological recovery. Nature.

[44] Xiong LL, Xue LL, Du RL, Niu RZ, Chen L, Chen J (2021). Single-cell RNA sequencing reveals B cell-related molecular biomarkers for Alzheimer’s disease. Exp Mol Med.

[45] Keren-Shaul H, Spinrad A, Weiner A, Matcovitch-Natan O, Dvir-Szternfeld R, Ulland TK (2017). A Unique Microglia Type Associated with Restricting Development of Alzheimer’s Disease. Cell.

[46] Sun XY, Yu XL, Zhu J, Li LJ, Zhang L, Huang YR (2023). Fc effector of anti-Aβ antibody induces synapse loss and cognitive deficits in Alzheimer’s disease-like mouse model. Signal Transduct Target Therapy.

[47] Zhang Y, Fung ITH, Sankar P, Chen X, Robison LS, Ye L et al. Depletion of NK Cells Improves Cognitive Function in the Alzheimer Disease Mouse Model. Journal of immunology (Baltimore, Md: 1950). 2020; 205(2): 502 – 10. 10.4049/jimmunol.2000037 PMC7343613 32503894

[48] Lu Y, Li K, Hu Y, Wang X (2021). Expression of Immune Related Genes and Possible Regulatory Mechanisms in Alzheimer’s Disease. Front Immunol.

[49] Herisson F, Frodermann V, Courties G, Rohde D, Sun Y, Vandoorne K (2018). Direct vascular channels connect skull bone marrow and the brain surface enabling myeloid cell migration. Nat Neurosci.

[50] van de Haar HJ, Burgmans S, Hofman PA, Verhey FR, Jansen JF, Backes WH (2015). Blood-brain barrier impairment in dementia: current and future in vivo assessments. Neurosci Biobehav Rev.

[51] Dong Y, Lagarde J, Xicota L, Corne H, Chantran Y, Chaigneau T (2018). Neutrophil hyperactivation correlates with Alzheimer’s disease progression. Ann Neurol.

[52] Chen M, Lam BK, Kanaoka Y, Nigrovic PA, Audoly LP, Austen KF (2006). Neutrophil-derived leukotriene B4 is required for inflammatory arthritis. J Exp Med.

[53] Grammas P (2000). A damaged microcirculation contributes to neuronal cell death in Alzheimer’s disease. Neurobiol Aging.

[54] Stroncek DF (2007). Neutrophil-specific antigen HNA-2a, NB1 glycoprotein, and CD177. Curr Opin Hematol.

[55] Cruz Hernández JC, Bracko O, Kersbergen CJ, Muse V, Haft-Javaherian M, Berg M (2019). Neutrophil adhesion in brain capillaries reduces cortical blood flow and impairs memory function in Alzheimer’s disease mouse models. Nat Neurosci.

[56] DiStasi MR, Ley K (2009). Opening the flood-gates: how neutrophil-endothelial interactions regulate permeability. Trends Immunol.

[57] Neeli I, Dwivedi N, Khan S, Radic M (2009). Regulation of extracellular chromatin release from neutrophils. J Innate Immun.

[58] Gautam N, Herwald H, Hedqvist P, Lindbom L (2000). Signaling via beta(2) integrins triggers neutrophil-dependent alteration in endothelial barrier function. J Exp Med.

[59] Ley K, Laudanna C, Cybulsky MI, Nourshargh S (2007). Getting to the site of inflammation: the leukocyte adhesion cascade updated. Nat Rev Immunol.

[60] Zlokovic BV (2011). Neurovascular pathways to neurodegeneration in Alzheimer’s disease and other disorders. Nat Rev Neurosci.

[61] Giri R, Shen Y, Stins M, Du Yan S, Schmidt AM, Stern D (2000). beta-amyloid-induced migration of monocytes across human brain endothelial cells involves RAGE and PECAM-1. Am J Physiol Cell Physiol.

[62] Yoon JH, Shin P, Joo J, Kim GS, Oh WY, Jeong Y (2022). Increased capillary stalling is associated with endothelial glycocalyx loss in subcortical vascular dementia. J Cereb Blood flow Metabolism: Official J Int Soc Cereb Blood Flow Metabolism.

[63] Li J, Kumari T, Barazia A, Jha V, Jeong SY, Olson A et al. Neutrophil DREAM promotes neutrophil recruitment in vascular inflammation. J Exp Med. 2022; 219(1). 10.1084/jem.20211083 PMC8719643 34751735

[64] Sweeney MD, Kisler K, Montagne A, Toga AW, Zlokovic BV (2018). The role of brain vasculature in neurodegenerative disorders. Nat Neurosci.

[65] Bracko O, Njiru BN, Swallow M, Ali M, Haft-Javaherian M, Schaffer CB (2020). Increasing cerebral blood flow improves cognition into late stages in Alzheimer’s disease mice. J Cereb Blood flow Metabolism: Official J Int Soc Cereb Blood Flow Metabolism.

[66] Gherardelli C, Cisternas P, Vera-Salazar RF, Mendez-Orellana C, Inestrosa NC (2022). Age- and Sex-Associated glucose metabolism decline in a mouse model of Alzheimer’s Disease. J Alzheimer’s Disease: JAD.

[67] Rossi B, Angiari S, Zenaro E, Budui SL, Constantin G. Vascular inflammation in central nervous system diseases: adhesion receptors controlling leukocyte-endothelial interactions. J Leukoc Biol 2011; 89(4): 539 – 56.[. 10.1189/jlb.0710432 21169520

[68] Carestia A, Kaufman T, Rivadeneyra L, Landoni VI, Pozner RG, Negrotto S (2016). Mediators and molecular pathways involved in the regulation of neutrophil extracellular trap formation mediated by activated platelets. J Leukoc Biol.

[69] Bu XL, Yao XQ, Jiao SS, Zeng F, Liu YH, Xiang Y (2015). A study on the association between infectious burden and Alzheimer’s disease. Eur J Neurol.

[70] Davydova TV, Fomina VG, Voskresenskaya NI, Doronina OA (2003). Phagocytic activity and state of bactericidal systems in polymorphonuclear leukocytes from patients with Alzheimer’s disease. Bull Exp Biol Med.

[71] Le Page A, Lamoureux J, Bourgade K, Frost EH, Pawelec G, Witkowski JM (2017). Polymorphonuclear Neutrophil functions are differentially altered in amnestic mild cognitive impairment and mild Alzheimer’s Disease patients. J Alzheimer’s Disease: JAD.

[72] Vitte J, Michel BF, Bongrand P, Gastaut JL (2004). Oxidative stress level in circulating neutrophils is linked to neurodegenerative diseases. J Clin Immunol.

[73] Bawa KK, Krance SH, Herrmann N, Cogo-Moreira H, Ouk M, Yu D (2020). A peripheral neutrophil-related inflammatory factor predicts a decline in executive function in mild Alzheimer’s disease. J Neuroinflamm.

[74] Scali C, Prosperi C, Bracco L, Piccini C, Baronti R, Ginestroni A (2002). Neutrophils CD11b and fibroblasts PGE(2) are elevated in Alzheimer’s disease. Neurobiol Aging.

[75] Naudé PJ, Nyakas C, Eiden LE, Ait-Ali D, van der Heide R, Engelborghs S (2012). Lipocalin 2: novel component of proinflammatory signaling in Alzheimer’s disease. FASEB Journal: Official Publication Federation Am Soc Experimental Biology.

[76] Choi J, Lee HW, Suk K (2011). Increased plasma levels of lipocalin 2 in mild cognitive impairment. J Neurol Sci.

[77] Hermann P, Villar-Piqué A, Schmitz M, Schmidt C, Varges D, Goebel S (2022). Plasma lipocalin 2 in Alzheimer’s disease: potential utility in the differential diagnosis and relationship with other biomarkers. Alzheimers Res Ther.

[78] Kang H, Shin HJ, An HS, Jin Z, Lee JY, Lee J (2021). Role of Lipocalin-2 in amyloid-Beta Oligomer-Induced Mouse Model of Alzheimer’s Disease. Antioxid (Basel Switzerland).

[79] Eruysal E, Ravdin L, Kamel H, Iadecola C, Ishii M (2019). Plasma lipocalin-2 levels in the preclinical stage of Alzheimer’s disease.

[80] Tzikas S, Schlak D, Sopova K, Gatsiou A, Stakos D, Stamatelopoulos K (2014). Increased myeloperoxidase plasma levels in patients with Alzheimer’s disease. J Alzheimer’s Disease: JAD.

[81] Gellhaar S, Sunnemark D, Eriksson H, Olson L, Galter D (2017). Myeloperoxidase-immunoreactive cells are significantly increased in brain areas affected by neurodegeneration in Parkinson’s and Alzheimer’s disease. Cell Tissue Res.

[82] Chou OHI, Zhou J, Li L, Chan JSK, Satti DI, Chou VHC (2023). The Association between Neutrophil-Lymphocyte ratio and variability with New-Onset Dementia: a Population-based Cohort Study. J Alzheimer’s Disease: JAD.

[83] Evlice A, Sanli ZS, Boz PB (2023). The importance of Vitamin-D and neutrophil-lymphocyte ratio for Alzheimer’s Disease. Pakistan J Med Sci.

[84] Dong X, Nao J, Shi J, Zheng D (2019). Predictive value of routine peripheral blood biomarkers in Alzheimer’s Disease. Front Aging Neurosci.

[85] Kuyumcu ME, Yesil Y, Oztürk ZA, Kizilarslanoğlu C, Etgül S, Halil M (2012). The evaluation of neutrophil-lymphocyte ratio in Alzheimer’s disease. Dement Geriatr Cogn Disord.

[86] Kara SP, Altunan B, Unal A (2022). Investigation of the peripheral inflammation (neutrophil-lymphocyte ratio) in two neurodegenerative diseases of the central nervous system. Neurol Sciences: Official J Italian Neurol Soc Italian Soc Clin Neurophysiol.

[87] An P, Zhou X, Du Y, Zhao J, Song A, Liu H (2019). Association of Neutrophil-Lymphocyte ratio with mild cognitive impairment in Elderly Chinese adults: a case-control study. Curr Alzheimer Res.

[88] Papayannopoulos V (2018). Neutrophil extracellular traps in immunity and disease. Nat Rev Immunol.

[89] Adrover JM, McDowell SAC, He XY, Quail DF, Egeblad M (2023). NETworking with cancer: the bidirectional interplay between cancer and neutrophil extracellular traps. Cancer Cell.

[90] Döring Y, Libby P, Soehnlein O (2020). Neutrophil Extracellular traps Participate in Cardiovascular diseases: recent experimental and clinical insights. Circul Res.

[91] Wigerblad G, Kaplan MJ (2023). Neutrophil extracellular traps in systemic autoimmune and autoinflammatory diseases. Nat Rev Immunol.

[92] Schulz C, Gabriel G, von Köckritz-Blickwede M (2020). Detrimental role of Neutrophil Extracellular traps during Dengue Virus infection. Trends Immunol.

[93] Rada B. Neutrophil Extracellular Traps. Methods in molecular biology (Clifton, NJ). 2019; 1982: 517 – 28. 10.1007/978-1-4939-9424-3_31 PMC6874304 31172493

[94] Papayannopoulos V, Metzler KD, Hakkim A, Zychlinsky A (2010). Neutrophil elastase and myeloperoxidase regulate the formation of neutrophil extracellular traps. J Cell Biol.

[95] Kebir H, Kreymborg K, Ifergan I, Dodelet-Devillers A, Cayrol R, Bernard M (2007). Human TH17 lymphocytes promote blood-brain barrier disruption and central nervous system inflammation. Nat Med.

[96] Sacks D, Baxter B, Campbell BCV, Carpenter JS, Cognard C, Dippel D (2018). Multisociety Consensus Quality Improvement revised Consensus Statement for Endovascular Therapy of Acute ischemic stroke. Int J Stroke: Official J Int Stroke Soc.

[97] Keshari RS, Jyoti A, Dubey M, Kothari N, Kohli M, Bogra J (2012). Cytokines induced neutrophil extracellular traps formation: implication for the inflammatory disease condition. PLoS ONE.

[98] Vukic V, Callaghan D, Walker D, Lue LF, Liu QY, Couraud PO (2009). Expression of inflammatory genes induced by beta-amyloid peptides in human brain endothelial cells and in Alzheimer’s brain is mediated by the JNK-AP1 signaling pathway. Neurobiol Dis.

[99] Garcia-Romo GS, Caielli S, Vega B, Connolly J, Allantaz F, Xu Z (2011). Netting neutrophils are major inducers of type I IFN production in pediatric systemic lupus erythematosus. Sci Transl Med.

[100] Kessenbrock K, Krumbholz M, Schönermarck U, Back W, Gross WL, Werb Z (2009). Netting neutrophils in autoimmune small-vessel vasculitis. Nat Med.

[101] Venereau E, Casalgrandi M, Schiraldi M, Antoine DJ, Cattaneo A, De Marchis F (2012). Mutually exclusive redox forms of HMGB1 promote cell recruitment or proinflammatory cytokine release. J Exp Med.

[102] Maugeri N, Campana L, Gavina M, Covino C, De Metrio M, Panciroli C (2014). Activated platelets present high mobility group box 1 to neutrophils, inducing autophagy and promoting the extrusion of neutrophil extracellular traps. J Thromb Haemostasis: JTH.

[103] Wolska N, Celikag M, Failla AV, Tarafdar A, Renné T, Torti M (2023). Human platelets release amyloid peptides β(1–40) and β(1–42) in response to haemostatic, immune, and hypoxic stimuli. Res Pract Thromb Haemostasis.

[104] Clark SR, Ma AC, Tavener SA, McDonald B, Goodarzi Z, Kelly MM (2007). Platelet TLR4 activates neutrophil extracellular traps to ensnare bacteria in septic blood. Nat Med.

[105] Kim SW, Lee JK. Role of HMGB1 in the interplay between NETosis and thrombosis in ischemic stroke: a review. Cells. 2020; 9(8). 10.3390/cells9081794 PMC7464684 32731558

[106] Tadie JM, Bae HB, Jiang S, Park DW, Bell CP, Yang H (2013). HMGB1 promotes neutrophil extracellular trap formation through interactions with toll-like receptor 4. Am J Physiol Lung Cell Mol Physiol.

[107] Ren J, He J, Zhang H, Xia Y, Hu Z, Loughran P (2021). Platelet TLR4-ERK5 Axis facilitates NET-Mediated capturing of circulating Tumor cells and distant metastasis after Surgical stress. Cancer Res.

[108] Boyer MJ, Kimura Y, Akiyama T, Baggett AY, Preston KJ, Scalia R (2020). Endothelial cell-derived extracellular vesicles alter vascular smooth muscle cell phenotype through high-mobility group box proteins. J Extracell Vesicles.

[109] Fiuza C, Bustin M, Talwar S, Tropea M, Gerstenberger E, Shelhamer JH (2003). Inflammation-promoting activity of HMGB1 on human microvascular endothelial cells. Blood.

[110] Dekens DW, Naudé PJ, Engelborghs S, Vermeiren Y, Van Dam D, Oude Voshaar RC (2017). Neutrophil Gelatinase-Associated Lipocalin and its receptors in Alzheimer’s Disease (AD) brain regions: Differential findings in AD with and without Depression. J Alzheimer’s Disease: JAD.

[111] Adler O, Zait Y, Cohen N, Blazquez R, Doron H, Monteran L (2023). Reciprocal interactions between innate immune cells and astrocytes facilitate neuroinflammation and brain metastasis via lipocalin-2. Nat cancer.

[112] Wu CY, Bawa KK, Ouk M, Leung N, Yu D, Lanctôt KL (2020). Neutrophil activation in Alzheimer’s disease and mild cognitive impairment: a systematic review and meta-analysis of protein markers in blood and cerebrospinal fluid. Ageing Res Rev.

[113] Dietrich MO, Spuch C, Antequera D, Rodal I, de Yébenes JG, Molina JA (2008). Megalin mediates the transport of leptin across the blood-CSF barrier. Neurobiol Aging.

[114] Aratani Y, Myeloperoxidase (2018). Its role for host defense, inflammation, and neutrophil function. Arch Biochem Biophys.

[115] Üllen A, Singewald E, Konya V, Fauler G, Reicher H, Nusshold C (2013). Myeloperoxidase-derived oxidants induce blood-brain barrier dysfunction in vitro and in vivo. PLoS ONE.

[116] Smyth LCD, Murray HC, Hill M, van Leeuwen E, Highet B, Magon NJ (2022). Neutrophil-vascular interactions drive myeloperoxidase accumulation in the brain in Alzheimer’s disease. Acta Neuropathol Commun.

[117] Mañucat-Tan NB, Chowdhury A, Cataldi R, Abdullah RZ, Kumita JR, Wyatt AR (2023). Hypochlorite-induced oxidation promotes aggregation and reduces toxicity of amyloid beta 1–42. Redox Biol.

[118] Eiserich JP, Baldus S, Brennan ML, Ma W, Zhang C, Tousson A (2002). Myeloperoxidase, a leukocyte-derived vascular NO oxidase.

[119] Maki RA, Tyurin VA, Lyon RC, Hamilton RL, DeKosky ST, Kagan VE (2009). Aberrant expression of myeloperoxidase in astrocytes promotes phospholipid oxidation and memory deficits in a mouse model of Alzheimer disease. J Biol Chem.

[120] Rembach A, Watt AD, Wilson WJ, Rainey-Smith S, Ellis KA, Rowe CC (2014). An increased neutrophil-lymphocyte ratio in Alzheimer’s disease is a function of age and is weakly correlated with neocortical amyloid accumulation. J Neuroimmunol.

[121] Huang LT, Zhang CP, Wang YB, Wang JH (2022). Association of Peripheral Blood Cell Profile with Alzheimer’s Disease: a Meta-analysis. Front Aging Neurosci.

[122] Iliff JJ, Wang M, Liao Y, Plogg BA, Peng W, Gundersen GA (2012). A paravascular pathway facilitates CSF flow through the brain parenchyma and the clearance of interstitial solutes, including amyloid β. Sci Transl Med.

[123] Weller RO, Djuanda E, Yow HY, Carare RO (2009). Lymphatic drainage of the brain and the pathophysiology of neurological disease. Acta Neuropathol.

[124] Togo T, Akiyama H, Iseki E, Kondo H, Ikeda K, Kato M (2002). Occurrence of T cells in the brain of Alzheimer’s disease and other neurological diseases. J Neuroimmunol.

[125] Larbi A, Pawelec G, Witkowski JM, Schipper HM, Derhovanessian E, Goldeck D (2009). Dramatic shifts in circulating CD4 but not CD8 T cell subsets in mild Alzheimer’s disease. J Alzheimer’s Disease: JAD.

[126] Oberstein TJ, Taha L, Spitzer P, Hellstern J, Herrmann M, Kornhuber J (2018). Imbalance of circulating T(h)17 and Regulatory T Cells in Alzheimer’s Disease: a Case Control Study. Front Immunol.

[127] Saresella M, Calabrese E, Marventano I, Piancone F, Gatti A, Alberoni M (2011). Increased activity of Th-17 and Th-9 lymphocytes and a skewing of the post-thymic differentiation pathway are seen in Alzheimer’s disease. Brain Behav Immun.

[128] Dansokho C, Ait Ahmed D, Aid S, Toly-Ndour C, Chaigneau T, Calle V (2016). Regulatory T cells delay disease progression in Alzheimer-like pathology. Brain.

[129] Lambracht-Washington D, Qu BX, Fu M, Anderson LD, Stüve O, Eagar TN (2011). DNA immunization against amyloid beta 42 has high potential as safe therapy for Alzheimer’s disease as it diminishes antigen-specific Th1 and Th17 cell proliferation. Cell Mol Neurobiol.

[130] Zhang J, Ke KF, Liu Z, Qiu YH, Peng YP (2013). Th17 cell-mediated neuroinflammation is involved in neurodegeneration of aβ1-42-induced Alzheimer’s disease model rats. PLoS ONE.

[131] Browne TC, McQuillan K, McManus RM, O’Reilly JA, Mills KH, Lynch MA. IFN-γ Production by amyloid β-specific Th1 cells promotes microglial activation and increases plaque burden in a mouse model of Alzheimer’s disease. Journal of immunology (Baltimore, Md: 1950). 2013; 190(5): 2241-51. 10.4049/jimmunol.1200947 23365075

[132] Zhang R, Miller RG, Madison C, Jin X, Honrada R, Harris W (2013). Systemic immune system alterations in early stages of Alzheimer’s disease. J Neuroimmunol.

[133] Richartz-Salzburger E, Batra A, Stransky E, Laske C, Köhler N, Bartels M (2007). Altered lymphocyte distribution in Alzheimer’s disease. J Psychiatr Res.

[134] Unger MS, Marschallinger J, Kaindl J, Klein B, Johnson M, Khundakar AA et al. Doublecortin expression in CD8 + T-cells and microglia at sites of amyloid-β plaques: A potential role in shaping plaque pathology? Alzheimer’s & dementia: the journal of the Alzheimer’s Association. 2018; 14(8): 1022–37. 10.1016/j.jalz.2018.02.017 29630865

[135] Merlini M, Kirabali T, Kulic L, Nitsch RM, Ferretti MT (2018). Extravascular CD3 + T cells in brains of Alzheimer Disease patients correlate with tau but not with amyloid Pathology: an immunohistochemical study. Neuro-degener Dis.

[136] Unger MS, Li E, Scharnagl L, Poupardin R, Altendorfer B, Mrowetz H (2020). CD8(+) T-cells infiltrate Alzheimer’s disease brains and regulate neuronal- and synapse-related gene expression in APP/PS1 transgenic mice. Brain Behav Immun.

[137] Wu CT, Chu CI, Wang FY, Yang HY, Tseng WS, Chang CR (2022). A change of PD-1/PD-L1 expression on peripheral T cell subsets correlates with the different stages of Alzheimer’s Disease. Cell Bioscience.

[138] Baruch K, Deczkowska A, Rosenzweig N, Tsitsou-Kampeli A, Sharif AM, Matcovitch-Natan O (2016). PD-1 immune checkpoint blockade reduces pathology and improves memory in mouse models of Alzheimer’s disease. Nat Med.

[139] Rosenzweig N, Dvir-Szternfeld R, Tsitsou-Kampeli A, Keren-Shaul H, Ben-Yehuda H, Weill-Raynal P (2019). PD-1/PD-L1 checkpoint blockade harnesses monocyte-derived macrophages to combat cognitive impairment in a tauopathy mouse model. Nat Commun.

[140] Martin E, Amar M, Dalle C, Youssef I, Boucher C, Le Duigou C (2019). New role of P2 × 7 receptor in an Alzheimer’s disease mouse model. Mol Psychiatry.

[141] Rinne JO, Brooks DJ, Rossor MN, Fox NC, Bullock R, Klunk WE (2010). 11 C-PiB PET assessment of change in fibrillar amyloid-beta load in patients with Alzheimer’s disease treated with bapineuzumab: a phase 2, double-blind, placebo-controlled, ascending-dose study. Lancet Neurol.

[142] Rampe D, Wang L, Ringheim GE (2004). P2 × 7 receptor modulation of beta-amyloid- and LPS-induced cytokine secretion from human macrophages and microglia. J Neuroimmunol.

[143] Sanz JM, Chiozzi P, Ferrari D, Colaianna M, Idzko M, Falzoni S (2009). Activation of microglia by amyloid {beta} requires P2 × 7 receptor expression. J Immunol (Baltimore Md: 1950).

[144] Miossec P, Kolls JK (2012). Targeting IL-17 and TH17 cells in chronic inflammation. Nat Rev Drug Discovery.

[145] Zhang Y, Liu M, Sun H, Yin K (2015). Matrine improves cognitive impairment and modulates the balance of Th17/Treg cytokines in a rat model of Aβ1-42-induced Alzheimer’s disease. Central-European J Immunol.

[146] Korn T, Bettelli E, Gao W, Awasthi A, Jäger A, Strom TB (2007). IL-21 initiates an alternative pathway to induce proinflammatory T(H)17 cells. Nature.

[147] Brigas HC, Ribeiro M, Coelho JE, Gomes R, Gomez-Murcia V, Carvalho K (2021). IL-17 triggers the onset of cognitive and synaptic deficits in early stages of Alzheimer’s disease. Cell Rep.

[148] Cristiano C, Volpicelli F, Lippiello P, Buono B, Raucci F, Piccolo M (2019). Neutralization of IL-17 rescues amyloid-β-induced neuroinflammation and memory impairment. Br J Pharmacol.

[149] Vellecco V, Saviano A, Raucci F, Casillo GM, Mansour AA, Panza E (2023). Interleukin-17 (IL-17) triggers systemic inflammation, peripheral vascular dysfunction, and related prothrombotic state in a mouse model of Alzheimer’s disease. Pharmacol Res.

[150] Machhi J, Yeapuri P, Lu Y, Foster E, Chikhale R, Herskovitz J (2021). CD4 + effector T cells accelerate Alzheimer’s disease in mice. J Neuroinflamm.

[151] Vom Berg J, Prokop S, Miller KR, Obst J, Kälin RE, Lopategui-Cabezas I (2012). Inhibition of IL-12/IL-23 signaling reduces Alzheimer’s disease-like pathology and cognitive decline. Nat Med.

[152] Teng MW, Bowman EP, McElwee JJ, Smyth MJ, Casanova JL, Cooper AM (2015). IL-12 and IL-23 cytokines: from discovery to targeted therapies for immune-mediated inflammatory diseases. Nat Med.

[153] Sakaguchi S, Mikami N, Wing JB, Tanaka A, Ichiyama K, Ohkura N (2020). Regulatory T cells and human disease. Annu Rev Immunol.

[154] Ciccocioppo F, Lanuti P, Pierdomenico L, Simeone P, Bologna G, Ercolino E (2019). The characterization of Regulatory T-Cell profiles in Alzheimer’s Disease and multiple sclerosis. Sci Rep.

[155] Anderson KM, Olson KE, Estes KA, Flanagan K, Gendelman HE, Mosley RL (2014). Dual destructive and protective roles of adaptive immunity in neurodegenerative disorders. Translational Neurodegeneration.

[156] Le Page A, Garneau H, Dupuis G, Frost EH, Larbi A, Witkowski JM (2017). Differential phenotypes of Myeloid-Derived Suppressor and T Regulatory Cells and cytokine levels in amnestic mild cognitive impairment subjects compared to mild Alzheimer diseased patients. Front Immunol.

[157] Rosenkranz D, Weyer S, Tolosa E, Gaenslen A, Berg D, Leyhe T et al. Higher frequency of regulatory T cells in the elderly and increased suppressive activity in neurodegeneration. Journal of neuroimmunology. 2007; 188(1–2): 117 – 27. 10.1016/j.jneuroim.2007.05.011 17582512

[158] Baruch K, Rosenzweig N, Kertser A, Deczkowska A, Sharif AM, Spinrad A (2015). Breaking immune tolerance by targeting Foxp3(+) regulatory T cells mitigates Alzheimer’s disease pathology. Nat Commun.

[159] Yeapuri P, Machhi J, Lu Y, Abdelmoaty MM, Kadry R, Patel M (2023). Amyloid-β specific regulatory T cells attenuate Alzheimer’s disease pathobiology in APP/PS1 mice. Mol Neurodegeneration.

[160] Liston A, Dooley J, Yshii L (2022). Brain-resident regulatory T cells and their role in health and disease. Immunol Lett.

[161] Xie L, Choudhury GR, Winters A, Yang SH, Jin K (2015). Cerebral regulatory T cells restrain microglia/macrophage-mediated inflammatory responses via IL-10. Eur J Immunol.

[162] Faridar A, Vasquez M, Thome AD, Yin Z, Xuan H, Wang JH (2022). Ex vivo expanded human regulatory T cells modify neuroinflammation in a preclinical model of Alzheimer’s disease. Acta Neuropathol Commun.

[163] Yang H, Park SY, Baek H, Lee C, Chung G, Liu X (2022). Adoptive therapy with amyloid-β specific regulatory T cells alleviates Alzheimer’s disease. Theranostics.

[164] Kunis G, Baruch K, Miller O, Schwartz M (2015). Immunization with a myelin-derived Antigen activates the Brain’s Choroid Plexus for Recruitment of Immunoregulatory Cells to the CNS and attenuates Disease Progression in a mouse model of ALS. J Neuroscience: Official J Soc Neurosci.

[165] Leonard WJ, Lin JX (2023). Strategies to therapeutically modulate cytokine action. Nat Rev Drug Discovery.

[166] Nicoll JA, Wilkinson D, Holmes C, Steart P, Markham H, Weller RO (2003). Neuropathology of human Alzheimer disease after immunization with amyloid-beta peptide: a case report. Nat Med.

[167] Gilman S, Koller M, Black RS, Jenkins L, Griffith SG, Fox NC (2005). Clinical effects of Abeta immunization (AN1792) in patients with AD in an interrupted trial. Neurology.

[168] Farlow M, Arnold SE, van Dyck CH, Aisen PS, Snider BJ, Porsteinsson AP (2012). Safety and biomarker effects of solanezumab in patients with Alzheimer’s disease. Alzheimer’s Dement J Alzheimer’s Assoc.

[169] Doody RS, Thomas RG, Farlow M, Iwatsubo T, Vellas B, Joffe S (2014). Phase 3 trials of solanezumab for mild-to-moderate Alzheimer’s disease. N Engl J Med.

[170] Monsonego A, Zota V, Karni A, Krieger JI, Bar-Or A, Bitan G (2003). Increased T cell reactivity to amyloid beta protein in older humans and patients with Alzheimer disease. J Clin Investig.

[171] Toly-Ndour C, Lui G, Nunes MM, Bruley-Rosset M, Aucouturier P, Dorothée G. MHC-independent genetic factors control the magnitude of CD4 + T cell responses to amyloid-β peptide in mice through regulatory T cell-mediated inhibition. Journal of immunology (Baltimore, Md: 1950). 2011; 187(9): 4492 – 500. 10.4049/jimmunol.1003953 21949026

[172] Das P, Chapoval S, Howard V, David CS, Golde TE (2003). Immune responses against Abeta1-42 in HLA class II transgenic mice: implications for Abeta1-42 immune-mediated therapies. Neurobiol Aging.

[173] Rosset MB, Lui G, Dansokho C, Chaigneau T, Dorothée G (2015). Vaccine-induced Aβ-specific CD8 + T cells do not trigger autoimmune neuroinflammation in a murine model of Alzheimer’s disease. J Neuroinflamm.

[174] Koreth J, Matsuoka K, Kim HT, McDonough SM, Bindra B, Alyea EP (2011). Interleukin-2 and regulatory T cells in graft-versus-host disease. N Engl J Med.

[175] Alves S, Churlaud G, Audrain M, Michaelsen-Preusse K, Fol R, Souchet B (2017). Interleukin-2 improves amyloid pathology, synaptic failure and memory in Alzheimer’s disease mice. Brain.

[176] Eremenko E, Mittal K, Berner O, Kamenetsky N, Nemirovsky A, Elyahu Y (2019). BDNF-producing, amyloid β-specific CD4 T cells as targeted drug-delivery vehicles in Alzheimer’s disease. EBioMedicine.

[177] Shen Y, Wang H, Sun Q, Yao H, Keegan AP, Mullan M (2018). Increased plasma Beta-secretase 1 May Predict Conversion to Alzheimer’s Disease Dementia in individuals with mild cognitive impairment. Biol Psychiatry.

[178] Wu X, Shen Q, Chang H, Li J, Xing D (2022). Promoted CD4(+) T cell-derived IFN-γ/IL-10 by photobiomodulation therapy modulates neurogenesis to ameliorate cognitive deficits in APP/PS1 and 3xTg-AD mice. J Neuroinflamm.

[179] Ma X, Nakayamada S, Kubo S, Sakata K, Yamagata K, Miyazaki Y (2018). Expansion of T follicular helper-T helper 1 like cells through epigenetic regulation by signal transducer and activator of transcription factors. Ann Rheum Dis.

[180] Carey AJ, Tan CK, Ulett GC (2012). Infection-induced IL-10 and JAK-STAT: a review of the molecular circuitry controlling immune hyperactivity in response to pathogenic microbes. Jak-stat.

[181] Cunningham C (2013). Microglia and neurodegeneration: the role of systemic inflammation. Glia.

[182] Song L, Yang YT, Guo Q, Zhao XM (2022). Cellular transcriptional alterations of peripheral blood in Alzheimer’s disease. BMC Med.

[183] Feng W, Zhang Y, Ding S, Chen S, Wang T, Wang Z et al. B lymphocytes ameliorate Alzheimer’s disease-like neuropathology via interleukin-35. Brain, behavior, and immunity. 2023; 108: 16–31. 10.1016/j.bbi.2022.11.012 36427805

[184] Posner DA, Lee CY, Portet A, Clatworthy MR (2022). Humoral immunity at the brain borders in homeostasis. Curr Opin Immunol.

[185] Verthelyi D (2001). Sex hormones as immunomodulators in health and disease. Int Immunopharmacol.

[186] Zhou Y, Zhao W, Al-Muhtasib N, Rebeck GW (2015). APOE genotype alters immunoglobulin subtypes in Knock-In mice. J Alzheimer’s Disease: JAD.

[187] Zhang L, Xu J, Gao J, Chen P, Yin M, Zhao W (2019). Decreased immunoglobulin G in brain regions of elder female APOE4-TR mice accompany with Aβ accumulation.

[188] Söllvander S, Ekholm-Pettersson F, Brundin RM, Westman G, Kilander L, Paulie S (2015). Increased number of plasma B cells producing autoantibodies against Aβ42 protofibrils in Alzheimer’s Disease. J Alzheimer’s Disease: JAD.

[189] Colonna-Romano G, Bulati M, Aquino A, Pellicanò M, Vitello S, Lio D (2009). A double-negative (IgD-CD27-) B cell population is increased in the peripheral blood of elderly people. Mech Ageing Dev.

[190] Bulati M, Buffa S, Candore G, Caruso C, Dunn-Walters DK, Pellicanò M (2011). B cells and immunosenescence: a focus on IgG + IgD-CD27- (DN) B cells in aged humans. Ageing Res Rev.

[191] Bulati M, Buffa S, Martorana A, Gervasi F, Camarda C, Azzarello DM (2015). Double negative (IgG + IgD-CD27-) B cells are increased in a cohort of moderate-severe Alzheimer’s disease patients and show a pro-inflammatory trafficking receptor phenotype. J Alzheimer’s Disease: JAD.

[192] Gupta S, Su H, Bi R, Agrawal S, Gollapudi S (2005). Life and death of lymphocytes: a role in immunesenescence.

[193] Zhang QQ, Jiang T, Gu LZ, Zhu XC, Zhao HD, Gao Q (2016). Common polymorphisms within QPCT gene are Associated with the susceptibility of Schizophrenia in a Han Chinese Population. Mol Neurobiol.

[194] Madera-Salcedo IK, Sánchez-Hernández BE, Svyryd Y, Esquivel-Velázquez M, Rodríguez-Rodríguez N, Trejo-Zambrano MI et al. PPP2R2B hypermethylation causes acquired apoptosis deficiency in systemic autoimmune diseases. JCI Insight. 2019; 5(16). 10.1172/jci.insight.126457 PMC6777834 31335320

[195] Sun J, Wang Y (2020). KIR3DL2 in cutaneous T-cell lymphoma: from a promising biomarker to a potential therapeutic target. Br J Dermatol.

[196] Mittal K, Eremenko E, Berner O, Elyahu Y, Strominger I, Apelblat D (2019). CD4 T cells induce a subset of MHCII-Expressing microglia that attenuates Alzheimer Pathology. iScience.

[197] Marsh SE, Abud EM, Lakatos A, Karimzadeh A, Yeung ST, Davtyan H (2016). The adaptive immune system restrains Alzheimer’s disease pathogenesis by modulating microglial function. Proc Natl Acad Sci USA.

[198] Park JC, Noh J, Jang S, Kim KH, Choi H, Lee D (2022). Association of B cell profile and receptor repertoire with the progression of Alzheimer’s disease. Cell Rep.

[199] Kim K, Wang X, Ragonnaud E, Bodogai M, Illouz T, DeLuca M (2021). Therapeutic B-cell depletion reverses progression of Alzheimer’s disease. Nat Commun.

[200] Agrawal S, Abud EM, Snigdha S, Agrawal A (2018). IgM response against amyloid-beta in aging: a potential peripheral protective mechanism. Alzheimers Res Ther.

[201] Baulch JE, Acharya MM, Agrawal S, Apodaca LA, Monteiro C, Agrawal A (2020). Immune and inflammatory determinants underlying Alzheimer’s Disease Pathology. J Neuroimmune Pharmacology: Official J Soc NeuroImmune Pharmacol.

[202] Kishida T, Hiromura Y, Shin-Ya M, Asada H, Kuriyama H, Sugai M (2007). IL-21 induces inhibitor of differentiation 2 and leads to complete abrogation of anaphylaxis in mice. J Immunol (Baltimore Md: 1950).

[203] Recher M, Berglund LJ, Avery DT, Cowan MJ, Gennery AR, Smart J (2011). IL-21 is the primary common γ chain-binding cytokine required for human B-cell differentiation in vivo. Blood.

[204] Spolski R, Leonard WJ (2014). Interleukin-21: a double-edged sword with therapeutic potential. Nat Rev Drug Discovery.

[205] Wekerle H (2017). B cells in multiple sclerosis. Autoimmunity.

[206] Pellicanò M, Bulati M, Buffa S, Barbagallo M, Di Prima A, Misiano G (2010). Systemic immune responses in Alzheimer’s disease: in vitro mononuclear cell activation and cytokine production. J Alzheimer’s Disease: JAD.

[207] Sallusto F, Impellizzieri D, Basso C, Laroni A, Uccelli A, Lanzavecchia A (2012). T-cell trafficking in the central nervous system. Immunol Rev.

[208] McNamee EN, Masterson JC, Jedlicka P, Collins CB, Williams IR, Rivera-Nieves J (2013). Ectopic lymphoid tissue alters the chemokine gradient, increases lymphocyte retention and exacerbates murine ileitis. Gut.

[209] Müller G, Lipp M (2003). Shaping up adaptive immunity: the impact of CCR7 and CXCR5 on lymphocyte trafficking. Microcirculation (New York NY: 1994).

[210] Louveau A, Smirnov I, Keyes TJ, Eccles JD, Rouhani SJ, Peske JD (2015). Structural and functional features of central nervous system lymphatic vessels. Nature.

[211] Gylys KH, Fein JA, Tan AM, Cole GM (2003). Apolipoprotein E enhances uptake of soluble but not aggregated amyloid-beta protein into synaptic terminals. J Neurochem.

[212] Albus A, Gold M, Bach JP, Burg-Roderfeld M, Jördens M, Kirchhein Y (2018). Extending the functional characteristics of naturally occurring autoantibodies against β-Amyloid, prion protein and α-Synuclein. PLoS ONE.

[213] Huang YR, Xie XX, Ji M, Yu XL, Zhu J, Zhang LX (2019). Naturally occurring autoantibodies against α-synuclein rescues memory and motor deficits and attenuates α-synuclein pathology in mouse model of Parkinson’s disease. Neurobiol Dis.

[214] Morgan D, Diamond DM, Gottschall PE, Ugen KE, Dickey C, Hardy J (2000). A beta peptide vaccination prevents memory loss in an animal model of Alzheimer’s disease. Nature.

[215] Mruthinti S, Buccafusco JJ, Hill WD, Waller JL, Jackson TW, Zamrini EY (2004). Autoimmunity in Alzheimer’s disease: increased levels of circulating IgGs binding Abeta and RAGE peptides. Neurobiol Aging.

[216] Bergen AA, Kaing S, ten Brink JB, Gorgels TG, Janssen SF (2015). Gene expression and functional annotation of human choroid plexus epithelium failure in Alzheimer’s disease. BMC Genomics.

[217] Dodel R, Balakrishnan K, Keyvani K, Deuster O, Neff F, Andrei-Selmer LC (2011). Naturally occurring autoantibodies against beta-amyloid: investigating their role in transgenic animal and in vitro models of Alzheimer’s disease. J Neuroscience: Official J Soc Neurosci.

[218] McLaurin J, Cecal R, Kierstead ME, Tian X, Phinney AL, Manea M (2002). Therapeutically effective antibodies against amyloid-beta peptide target amyloid-beta residues 4–10 and inhibit cytotoxicity and fibrillogenesis. Nat Med.

[219] Holmes C, Boche D, Wilkinson D, Yadegarfar G, Hopkins V, Bayer A (2008). Long-term effects of Abeta42 immunisation in Alzheimer’s disease: follow-up of a randomised, placebo-controlled phase I trial. Lancet (London England).

[220] Weksler ME, Relkin N, Turkenich R, LaRusse S, Zhou L, Szabo P (2002). Patients with Alzheimer disease have lower levels of serum anti-amyloid peptide antibodies than healthy elderly individuals. Exp Gerontol.

[221] Brettschneider S, Morgenthaler NG, Teipel SJ, Fischer-Schulz C, Bürger K, Dodel R (2005). Decreased serum amyloid beta(1–42) autoantibody levels in Alzheimer’s disease, determined by a newly developed immuno-precipitation assay with radiolabeled amyloid beta(1–42) peptide. Biol Psychiatry.

[222] Du Y, Dodel R, Hampel H, Buerger K, Lin S, Eastwood B (2001). Reduced levels of amyloid beta-peptide antibody in Alzheimer disease. Neurology.

[223] Moir RD, Tseitlin KA, Soscia S, Hyman BT, Irizarry MC, Tanzi RE (2005). Autoantibodies to redox-modified oligomeric abeta are attenuated in the plasma of Alzheimer’s disease patients. J Biol Chem.

[224] Qu BX, Gong Y, Moore C, Fu M, German DC, Chang LY et al. Beta-amyloid auto-antibodies are reduced in Alzheimer’s disease. Journal of neuroimmunology. 2014; 274(1–2): 168 – 73. 10.1016/j.jneuroim.2014.06.017 PMC4410718 25022335

[225] Jianping L, Zhibing Y, Wei Q, Zhikai C, Jie X, Jinbiao L (2006). Low avidity and level of serum anti-abeta antibodies in Alzheimer disease. Alzheimer Dis Assoc Disord.

[226] Song MS, Mook-Jung I, Lee HJ, Min JY, Park MH (2007). Serum anti-amyloid-beta antibodies and Alzheimer’s disease in elderly Korean patients. J Int Med Res.

[227] Sohn JH, So JO, Hong HJ, Kim JW, Na DR, Kim M (2009). Identification of autoantibody against beta-amyloid peptide in the serum of elderly. Front Bioscience (Landmark Edition).

[228] Nath A, Hall E, Tuzova M, Dobbs M, Jons M, Anderson C (2003). Autoantibodies to amyloid beta-peptide (abeta) are increased in Alzheimer’s disease patients and abeta antibodies can enhance Abeta neurotoxicity: implications for disease pathogenesis and vaccine development. Neuromol Med.

[229] Gruden MA, Davudova TB, Malisauskas M, Zamotin VV, Sewell RD, Voskresenskaya NI (2004). Autoimmune responses to amyloid structures of Abeta(25–35) peptide and human lysozyme in the serum of patients with progressive Alzheimer’s disease. Dement Geriatr Cogn Disord.

[230] Gruden, Davidova, Malisauskas M, Sewell RD, Voskresenskaya, Wilhelm K (2007). Differential neuroimmune markers to the onset of Alzheimer’s disease neurodegeneration and dementia: autoantibodies to Abeta((25–35)) oligomers, S100b and neurotransmitters. J Neuroimmunol.

[231] Gustaw KA, Garrett MR, Lee HG, Castellani RJ, Zagorski MG, Prakasam A (2008). Antigen-antibody dissociation in Alzheimer disease: a novel approach to diagnosis. J Neurochem.

[232] Hyman BT, Smith C, Buldyrev I, Whelan C, Brown H, Tang MX (2001). Autoantibodies to amyloid-beta and Alzheimer’s disease. Ann Neurol.

[233] Baril L, Nicolas L, Croisile B, Crozier P, Hessler C, Sassolas A (2004). Immune response to abeta-peptides in peripheral blood from patients with Alzheimer’s disease and control subjects. Neurosci Lett.

[234] Xu W, Kawarabayashi T, Matsubara E, Deguchi K, Murakami T, Harigaya Y (2008). Plasma antibodies to Abeta40 and Abeta42 in patients with Alzheimer’s disease and normal controls. Brain Res.

[235] Cairns NJ, Lee VM, Trojanowski JQ (2004). The cytoskeleton in neurodegenerative diseases. J Pathol.

[236] Illouz T, Madar R, Hirsh T, Biragyn A, Okun E (2021). Induction of an effective anti-Amyloid-β humoral response in aged mice. Vaccine.

[237] van der Hoven J, van Hummel A, Przybyla M, Asih PR, Gajwani M, Feiten AF (2020). Contribution of endogenous antibodies to learning deficits and astrocytosis in human P301S mutant tau transgenic mice. Sci Rep.

[238] Maftei M, Thurm F, Schnack C, Tumani H, Otto M, Elbert T (2013). Increased levels of antigen-bound β-amyloid autoantibodies in serum and cerebrospinal fluid of Alzheimer’s disease patients. PLoS ONE.

[239] Dodel R, Rominger A, Bartenstein P, Barkhof F, Blennow K, Förster S (2013). Intravenous immunoglobulin for treatment of mild-to-moderate Alzheimer’s disease: a phase 2, randomised, double-blind, placebo-controlled, dose-finding trial. Lancet Neurol.

[240] St-Amour I, Paré I, Alata W, Coulombe K, Ringuette-Goulet C, Drouin-Ouellet J (2013). Brain bioavailability of human intravenous immunoglobulin and its transport through the murine blood-brain barrier. J Cereb Blood flow Metabolism: Official J Int Soc Cereb Blood Flow Metabolism.

[241] Traynelis SF, Wollmuth LP, McBain CJ, Menniti FS, Vance KM, Ogden KK (2010). Glutamate receptor ion channels: structure, regulation, and function. Pharmacol Rev.

[242] Parsons CG, Danysz W, Dekundy A, Pulte I (2013). Memantine and cholinesterase inhibitors: complementary mechanisms in the treatment of Alzheimer’s disease. Neurotox Res.

[243] Simma N, Bose T, Kahlfuss S, Mankiewicz J, Lowinus T, Lühder F (2014). NMDA-receptor antagonists block B-cell function but foster IL-10 production in BCR/CD40-activated B cells. Cell Communication Signaling: CCS.

[244] Michel T, Poli A, Cuapio A, Briquemont B, Iserentant G, Ollert M et al. Human CD56bright NK Cells: An Update. Journal of immunology (Baltimore, Md: 1950). 2016; 196(7): 2923-31. 10.4049/jimmunol.1502570 26994304

[245] Solerte SB, Cravello L, Ferrari E, Fioravanti M (2000). Overproduction of IFN-gamma and TNF-alpha from natural killer (NK) cells is associated with abnormal NK reactivity and cognitive derangement in Alzheimer’s disease. Ann N Y Acad Sci.

[246] Schindowski K, Peters J, Gorriz C, Schramm U, Weinandi T, Leutner S (2006). Apoptosis of CD4 + T and natural killer cells in Alzheimer’s disease. Pharmacopsychiatry.

[247] Masera RG, Prolo P, Sartori ML, Staurenghi A, Griot G, Ravizza L (2002). Mental deterioration correlates with response of natural killer (NK) cell activity to physiological modifiers in patients with short history of Alzheimer’s disease. Psychoneuroendocrinology.

[248] Liu Z, Li H, Pan S (2021). Discovery and Validation of key biomarkers based on Immune infiltrates in Alzheimer’s Disease. Front Genet.

[249] Rangaraju S, Dammer EB, Raza SA, Rathakrishnan P, Xiao H, Gao T (2018). Identification and therapeutic modulation of a pro-inflammatory subset of disease-associated-microglia in Alzheimer’s disease. Mol Neurodegeneration.

[250] Donahue JE, Flaherty SL, Johanson CE, Duncan JA, Silverberg GD, Miller MC (2006). RAGE, LRP-1, and amyloid-beta protein in Alzheimer’s disease. Acta Neuropathol.

[251] Kannan Y, Yu J, Raices RM, Seshadri S, Wei M, Caligiuri MA (2011). IκBζ augments IL-12- and IL-18-mediated IFN-γ production in human NK cells. Blood.

[252] Qi C, Liu F, Zhang W, Han Y, Zhang N, Liu Q (2022). Alzheimer’s disease alters the transcriptomic profile of natural killer cells at single-cell resolution. Front Immunol.

[253] Song TL, Nairismägi ML, Laurensia Y, Lim JQ, Tan J, Li ZM (2018). Oncogenic activation of the STAT3 pathway drives PD-L1 expression in natural killer/T-cell lymphoma. Blood.

[254] Martins LC, Rocha NP, Torres KC, Dos Santos RR, França GS, de Moraes EN et al. Disease-specific expression of the serotonin-receptor 5-HT(2 C) in natural killer cells in Alzheimer’s dementia. Journal of neuroimmunology. 2012; 251(1–2): 73 – 9. 10.1016/j.jneuroim.2012.06.003 22766135

[255] Lanari A, Amenta F, Silvestrelli G, Tomassoni D, Parnetti L (2006). Neurotransmitter deficits in behavioural and psychological symptoms of Alzheimer’s disease. Mech Ageing Dev.

[256] Liu Q, Sanai N, Jin WN, La Cava A, Van Kaer L, Shi FD (2016). Neural stem cells sustain natural killer cells that dictate recovery from brain inflammation. Nat Neurosci.

[257] Huntington ND, Legrand N, Alves NL, Jaron B, Weijer K, Plet A (2009). IL-15 trans-presentation promotes human NK cell development and differentiation in vivo. J Exp Med.

[258] Heppner FL, Ransohoff RM, Becher B (2015). Immune attack: the role of inflammation in Alzheimer disease. Nat Rev Neurosci.

[259] Wang J, Jin WS, Bu XL, Zeng F, Huang ZL, Li WW (2018). Physiological clearance of tau in the periphery and its therapeutic potential for tauopathies. Acta Neuropathol.

[260] Qosa H, Abuasal BS, Romero IA, Weksler B, Couraud PO, Keller JN (2014). Differences in amyloid-β clearance across mouse and human blood-brain barrier models: kinetic analysis and mechanistic modeling. Neuropharmacology.

[261] Butovsky O, Kunis G, Koronyo-Hamaoui M, Schwartz M (2007). Selective ablation of bone marrow-derived dendritic cells increases amyloid plaques in a mouse Alzheimer’s disease model. Eur J Neurosci.

[262] Schwartz M, Baruch K (2014). The resolution of neuroinflammation in neurodegeneration: leukocyte recruitment via the choroid plexus. EMBO J.

[263] Agrawal S, Anderson P, Durbeej M, van Rooijen N, Ivars F, Opdenakker G (2006). Dystroglycan is selectively cleaved at the parenchymal basement membrane at sites of leukocyte extravasation in experimental autoimmune encephalomyelitis. J Exp Med.

[264] Huang Y, Xu Z, Xiong S, Sun F, Qin G, Hu G (2018). Repopulated microglia are solely derived from the proliferation of residual microglia after acute depletion. Nat Neurosci.

[265] Sanchez-Mejias E, Navarro V, Jimenez S, Sanchez-Mico M, Sanchez-Varo R, Nuñez-Diaz C (2016). Soluble phospho-tau from Alzheimer’s disease hippocampus drives microglial degeneration. Acta Neuropathol.

[266] Cronk JC, Filiano AJ, Louveau A, Marin I, Marsh R, Ji E (2018). Peripherally derived macrophages can engraft the brain independent of irradiation and maintain an identity distinct from microglia. J Exp Med.

[267] Naert G, Rivest S (2013). A deficiency in CCR2 + monocytes: the hidden side of Alzheimer’s disease. J Mol Cell Biol.

[268] Prinz M, Priller J (2017). The role of peripheral immune cells in the CNS in steady state and disease. Nat Neurosci.

[269] Smit T, Ormel PR, Sluijs JA, Hulshof LA, Middeldorp J, de Witte LD (2022). Transcriptomic and functional analysis of Aβ(1–42) oligomer-stimulated human monocyte-derived microglia-like cells. Brain Behav Immun.

[270] Munawara U, Catanzaro M, Xu W, Tan C, Hirokawa K, Bosco N (2021). Hyperactivation of monocytes and macrophages in MCI patients contributes to the progression of Alzheimer’s disease.

[271] Fiala M, Zhang L, Gan X, Sherry B, Taub D, Graves MC (1998). Amyloid-beta induces chemokine secretion and monocyte migration across a human blood–brain barrier model. Mol Med (Cambridge Mass).

[272] Simard AR, Soulet D, Gowing G, Julien JP, Rivest S (2006). Bone marrow-derived microglia play a critical role in restricting senile plaque formation in Alzheimer’s disease. Neuron.

[273] Fani Maleki A, Cisbani G, Plante MM, Préfontaine P, Laflamme N, Gosselin J (2020). Muramyl dipeptide-mediated immunomodulation on monocyte subsets exerts therapeutic effects in a mouse model of Alzheimer’s disease. J Neuroinflamm.

[274] Saare M, Tserel L, Haljasmägi L, Taalberg E, Peet N, Eimre M (2020). Monocytes present age-related changes in phospholipid concentration and decreased energy metabolism. Aging Cell.

[275] Minhas PS, Latif-Hernandez A, McReynolds MR, Durairaj AS, Wang Q, Rubin A (2021). Restoring metabolism of myeloid cells reverses cognitive decline in ageing. Nature.

[276] Liu ZH, Bai YD, Yu ZY, Li HY, Liu J, Tan CR (2023). Improving blood Monocyte Energy Metabolism enhances its ability to phagocytose Amyloid-β and prevents Alzheimer’s Disease-Type Pathology and Cognitive deficits. Neurosci Bull.

[277] Ng A, Tam WW, Zhang MW, Ho CS, Husain SF, McIntyre RS (2018). IL-1β, IL-6, TNF- α and CRP in Elderly patients with Depression or Alzheimer’s disease: systematic review and Meta-analysis. Sci Rep.

[278] Griciuc A, Serrano-Pozo A, Parrado AR, Lesinski AN, Asselin CN, Mullin K (2013). Alzheimer’s disease risk gene CD33 inhibits microglial uptake of amyloid beta. Neuron.

[279] Reed-Geaghan EG, Savage JC, Hise AG, Landreth GE (2009). CD14 and toll-like receptors 2 and 4 are required for fibrillar A{beta}-stimulated microglial activation. J Neuroscience: Official J Soc Neurosci.

[280] Kanazawa M, Mori Y, Yoshihara K, Iwadate M, Suzuki S, Endoh Y (2004). Effect of PSK on the maturation of dendritic cells derived from human peripheral blood monocytes. Immunol Lett.

[281] Chen SH, He CY, Shen YY, Zeng GH, Tian DY, Cheng Y (2022). Polysaccharide Krestin prevents Alzheimer’s Disease-type Pathology and Cognitive deficits by enhancing monocyte Amyloid-β Processing. Neurosci Bull.

[282] Maurice T, Mustafa MH, Desrumaux C, Keller E, de la Naert G (2013). Intranasal formulation of erythropoietin (EPO) showed potent protective activity against amyloid toxicity in the Aβ₂₅₋₃₅ non-transgenic mouse model of Alzheimer’s disease. J Psychopharmacol (Oxford England).

[283] Brines M, Patel NS, Villa P, Brines C, Mennini T, De Paola M (2008). Nonerythropoietic, tissue-protective peptides derived from the tertiary structure of erythropoietin. Proc Natl Acad Sci USA.

[284] Al-Onaizi MA, Thériault P, Lecordier S, Prefontaine P, Rivest S, ElAli A (2022). Early monocyte modulation by the non-erythropoietic peptide ARA 290 decelerates AD-like pathology progression. Brain Behav Immun.

[285] Mintun MA, Lo AC, Duggan Evans C, Wessels AM, Ardayfio PA, Andersen SW (2021). Donanemab in Early Alzheimer’s Disease. N Engl J Med.

[286] van Dyck CH, Swanson CJ, Aisen P, Bateman RJ, Chen C, Gee M (2023). Lecanemab in Early Alzheimer’s Disease. N Engl J Med.

[287] Fantin A, Vieira JM, Gestri G, Denti L, Schwarz Q, Prykhozhij S (2010). Tissue macrophages act as cellular chaperones for vascular anastomosis downstream of VEGF-mediated endothelial tip cell induction. Blood.

[288] Uekawa K, Hattori Y, Ahn SJ, Seo J, Casey N, Anfray A (2023). Border-associated macrophages promote cerebral amyloid angiopathy and cognitive impairment through vascular oxidative stress. Mol Neurodegeneration.

[289] Cisternas P, Taylor X, Lasagna-Reeves CA (2019). The amyloid-tau-neuroinflammation Axis in the context of cerebral amyloid Angiopathy. Int J Mol Sci.

[290] Ketter N, Brashear HR, Bogert J, Di J, Miaux Y, Gass A (2017). Central Review of Amyloid-Related Imaging Abnormalities in two phase III clinical trials of Bapineuzumab in Mild-To-Moderate Alzheimer’s Disease patients. J Alzheimer’s Disease: JAD.

[291] Taylor X, Clark IM, Fitzgerald GJ, Oluoch H, Hole JT, DeMattos RB (2023). Amyloid-β (Aβ) immunotherapy induced microhemorrhages are associated with activated perivascular macrophages and peripheral monocyte recruitment in Alzheimer’s disease mice. Mol Neurodegeneration.

[292] Park L, Uekawa K, Garcia-Bonilla L, Koizumi K, Murphy M, Pistik R (2017). Brain perivascular macrophages initiate the neurovascular dysfunction of Alzheimer Aβ peptides. Circul Res.

[293] Pons V, Rivest S (2022). Targeting systemic Innate Immune cells as a Therapeutic Avenue for Alzheimer Disease. Pharmacol Rev.

